# Using Gene Essentiality and Synthetic Lethality Information to Correct Yeast and CHO Cell Genome-Scale Models

**DOI:** 10.3390/metabo5040536

**Published:** 2015-09-29

**Authors:** Ratul Chowdhury, Anupam Chowdhury, Costas D. Maranas

**Affiliations:** Department of Chemical Engineering, The Pennsylvania State University, University Park, Pennsylvania, PA 16802, USA; E-Mails: ratul@psu.edu (R.C.); axc1028@psu.edu (A.C.)

**Keywords:** synthetic lethality, yeast, Chinese Hamster Ovary, model curation

## Abstract

Essentiality (ES) and Synthetic Lethality (SL) information identify combination of genes whose deletion inhibits cell growth. This information is important for both identifying drug targets for tumor and pathogenic bacteria suppression and for flagging and avoiding gene deletions that are non-viable in biotechnology. In this study, we performed a comprehensive ES and SL analysis of two important eukaryotic models (*S. cerevisiae* and CHO cells) using a bilevel optimization approach introduced earlier. Information gleaned from this study is used to propose specific model changes to remedy inconsistent with data model predictions. Even for the highly curated Yeast 7.11 model we identified 50 changes (metabolic and GPR) leading to the correct prediction of an additional 28% of essential genes and 36% of synthetic lethals along with a 53% reduction in the erroneous identification of essential genes. Due to the paucity of mutant growth phenotype data only 12 changes were made for the CHO 1.2 model leading to an additional correctly predicted 11 essential and eight non-essential genes. Overall, we find that CHO 1.2 was 76% less accurate than the Yeast 7.11 metabolic model in predicting essential genes. Based on this analysis, 14 (single and double deletion) maximally informative experiments are suggested to improve the CHO cell model by using information from a mouse metabolic model. This analysis demonstrates the importance of single and multiple knockout phenotypes in assessing and improving model reconstructions. The advent of techniques such as CRISPR opens the door for the global assessment of eukaryotic models.

## 1. Introduction

Both budding yeast *S. cerevisiae* and Chinese Hamster Ovary (CHO) *C. griseus* are model cell lines for understanding metabolism in eukaryotes [1] as well as versatile bio-production hosts [2,3] for biofuels, biorenewables and proteins [4]. The earliest genome-scale metabolic model of yeast (*i*FF708 [5]) included 708 ORFs (10.7% of the total number of verified ORFs in yeast genome) and 1175 reactions with only two compartments (*i.e.*, mitochondria and cytosol). Subsequent efforts improved this model not only by including additional ORFs, metabolites and metabolic pathways, but also by integration of enzyme-localization information for compartmentalization (e.g., including peroxisome, nucleus, golgi apparatus, vacuole and endoplasmic reticulum) [6]. The latest consensus model version (Yeast 7.11 [7]) consists of 2,218 metabolites and 910 genes partitioned in 14 distinct compartments. A detailed comparison of the development of yeast genome-scale models is reviewed in [8,9]. CHO cells have emerged as the preferred cell line for recombinant proteins [10]. It has been shown that 70% of therapeutics production [11] worldwide is carried out in CHO cells thereby garnering over $30 billion in sales. An important consideration is for genetic engineering to avoid knockouts of lethal gene sets while designing high yielding strains [12] of commercial importance.

Essentiality and SL analyses [13,14] have been used to systematically assess the validity/accuracy of genome-scale flux models [8]. Essentiality and SL analyses refer to identifying sets of gene deletions (single, double and higher order thereof) that render the strain nonviable. Essentiality analysis identifies the list of genes, each of which when deleted *in silico*, limits the biomass flux to lower than 10% of its theoretical maximum. Whereas, SL analysis identifies the list of *in silico* gene pairs (and higher order) whose removal constrains the biomass flux to lower than the aforesaid essentiality criterion. These analyses serve the dual purpose of model refinement (by comparing with available *in vivo* knockout information) and prediction for identifying genes (or combination of genes) whose knockouts could potentially be lethal. The latter is particularly useful in strain engineering applications for avoiding synthetic lethal gene deletions. In earlier efforts, these analyses were used extensively to curate metabolic models of well-studied organisms such as *E. coli* [15,16] and *S.* *cerevisiae* [8]. Model improvement using network-embedded thermodynamic flux variability analyses to ascertain the directionality of reactions have been used by Martinez *et al.* [17]. Other related efforts include Stanford *et al.* [18] and Soh *et al.* [19], all of which aim to integrate thermodynamic information to curate the yeast genome scale models. In this study, we evaluate the accuracy of the latest genome-scale consensus model first for *S. cerevisiae* (Yeast 7.11) and thereby update it to *i*Sce926 and subsequently for *C. griseus* (CHO 1.2) with existing experimental measurements in terms of gene essentiality and synthetic lethality and propose a list of corrections and follow-up assessment of predicted gene deletions.

The proposed model modifications on Yeast 7.11 involve 50 literature-supported changes that improve the sensitivity, specificity of Yeast 7.11 by 2.66% and 20.4% and decrease the false viable rate (FVR) by 8.42% (see Appendix). They build upon the effort by Zomorrodi *et al.* [8] as they conserve four of earlier identified changes. ES and SL analyses are supplemented by auxotrophy information (for Precursor Identifier algorithm see Supplemental File S2) to help elucidate the cause (*i.e.*, nutrient or biomass precursor deficiency) for lethality. For CHO 1.2, we identified eight instances where model and experiment does not match. Upon supplementing this mismatched set with another 11 cases of model and experiment discrepancies from the mouse model [20], we suggested 14 additional (single, double and higher) gene deletion experiments (see Supplemental File S1) for maximally resolving mutant growth phenotypes in CHO cell lines.

## 2. Results and Discussion

### 2.1. S. Cerevisiae Model Yeast 7.11 Curation

**Figure 1:**
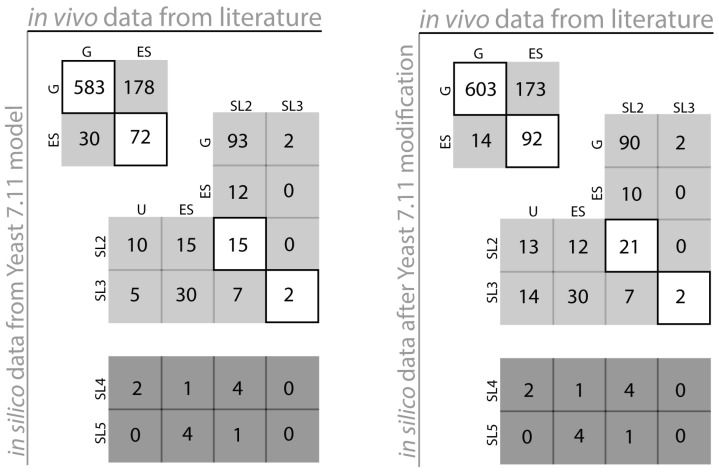
Yeast 7.11 model performance comparison (Yeast 7.11: left, *i*Sce926: right). The diagonal boxes represent points of *in silico-in vivo* match. The non-diagonal elements represent points of *in silico in vivo* inconsistency.

*In vivo* essentiality and synthetic lethality information were mostly obtained from gene deletion studies by Tong *et al.* [21]. Gene level essentiality analysis (see Table 1) and synthetic lethality predictions for Yeast 7.11 are shown in Figure 1. Resources used to assess *in silico* results included (i) the *Saccharomyces* Genome Database (SGD) [22], (ii) single gene deletion studies in minimal media on yeast strain S288C [23], (iii) viability analyses [24], (iv) data from The ORF Report [25,26,27] and analysis of protein encoded transmembrane segments in yeast [28] (See Supplemental File S3 for full gene lists and references of experimental evidence).

**Table 1:** Yeast 7.11 model statistics on Essentiality (ES) and Synthetic Lethality (SL).

	Parameter	Count
Essentiality information	Essential Reactions with GPRs	195
	Essential Reactions without GPRs	248
	Essential genes	151
Reaction level lethality	SL Pairs	70
	SL Triplets	21
	SL Quadruplets	11
	SL Quintuplets	NP^1^
Gene level lethality	SL Pairs	40
	SL Triplets	44
	SL Quadruplets	7
	SL Quintuplets	5

**^1^NP**: Not Performed.

Table 1 depicts the number of essential reactions (~13% of total reactions present in the model), essential genes (~16.5% of total genes in the model) and catalogs the number synthetic lethals with up to five simultaneous gene deletions identified by the SL finder for Yeast 7.11. Figure 1 pictorially classifies the agreement of *in silico* lethality information with *in vivo* gene deletion information (see Supplemental File S4: Table 2). The vertical axis refers to *in silico* predictions while the horizontal axis refers to *in vivo* data. The boxes in the diagonal are instances of compliance between model and experiment whereas boxes off the diagonal signify various modes of disagreement between *in silico* and *in vivo* data. For example, box ESG for the Yeast 7.11 encompasses 30 genes which are essential (ES) *in silico* but are non-essential (G) *in vivo* whereas box SL2ES refers to gene pairs that have been found to form a synthetic lethal pair *in silico* (SL2), however at least one of them is essential (ES) *in vivo.* Overall, our current model *i*Sce926 improves the sensitivity and specificity of Yeast 7.11 from 0.288 to 0.347 and 0.951 to 0.977 respectively and decreases the false viability rate (FVR) from 0.712 to 0.652 (see Appendix). Brief summaries of few of the proposed model modifications have been listed in Table 2, Table 3 and Table 4. Comprehensive information on the exact cause of disagreement between model and experiment can be found in Supplemental File S3. *In silico* analysis also generates results for deletion combinations unexplored so far. For example, box SL2U (see Figure 1) contains 10 *in silico* lethal gene pairs for which double deletion experiments have been unverified (U) so far in the literature. These unverified results often reveal non-intuitive lethal gene sets to be avoided while designing overproduction strains. Conversely, predicted viable synthetic lethals are prime candidates to be tested experimentally to assess the functionality of all the pathways present in the model. Absence of isozymes and alternate pathways in the model may also lead to discrepancies between *in silico* results and *in vivo* data. For example, gene *TYR1* (*YBR166C*) which is essential (ES) for tyrosine biosynthesis [29] according to the model, is found experimentally to be non-essential instead and found to form synthetic lethals (*i.e.*, a ESSL2 discrepancy). There is a single pathway for the formation of tyrosine in Yeast 7.11; however, chitin synthase (*CHS1*) [30,31], which is not present in the existing metabolic model, can rescue this mutant phenotype. Another such example involves gene *BAT2* (*YJR148W*) encoding branched-chain amino transferases in isoleucine, leucine and valine biosynthetic pathways [32]. *BAT2* forms an *in vivo* synthetic lethal pair with its paralog *BAT1* (*YHR208W*)*.* However, both have been identified as essential *in silico*. Reconciliation between model and experiment was achieved by adding the pretyrosine pathway [33] and allowing for the transport of α-keto-isovalerate across the mitochondrial membrane [34].

**Table 2:** Yeast 7.11 Model Modifications.

	No.	Model Modification	Improvement on Yeast 7.11	Remarks	Reference
**Addition of Reactions**	1	Addition of alpha-keto isovalerate (KIV) transport 3-methyl 2-oxobutanoate [m] ⬄ 3-methyl 2-oxobutanoate [c]	*BAT1* reconciled from ESG to GG *BAT1-BAT2* reconciled from ESSL2 to SL2SL2	The KIV transport provides an alternate path for cytosolic valine formation.	[25] [35]
	2	Mitochondrial acetyl-transferase activity of glycine CoA [m] + L-2 amino 3-oxobutanoate [m] ⬄ acetyl-CoA [m] + L-glycine [m] GPR: *YDL040C or YGR147C or YHR013C*	Correctly adds *NAT1, NAT2* and *ARD1* as GG	This adds a missing reaction and identifies the associated genes correctly as non-essential.	[36] [37]
**GPR modifications**	3	GPR modification for reaction r_0195 Old GPR: *((YBR126C and YDR074W and YMR261C) or (YML100W and YBR126C and YDR074W))* New GPR: *((YBR126C and YDR074W and YMR261C and YML100W) or YBR126C)*	*TPS1* gene is only essential in glucose media whereas both *TPS1* and *TPS2* genes are essential in galactose media reflected in old GPR *TPS2* gene is restored as a GG from an ESG	This shows a media dependent gene essentiality.	[38] [39] [40]
	4	GPR modification for reaction r_0995 Old GPR: *YDR023W or YHR011W* New GPR: *YDR023W or (YDR023W and YHR011W)*	*SES1* gene is corrected from GES to ESES *SES1-DIA1* is corrected from SL2ES and SL2G to ESES and GG cases respectively	The modification identifies *SES1* as the major isoform consistent to *in vivo* information.	[41] [42]
	5	GPR modification for reaction r_0250 Old GPR: *((YJR019C and YOR303W) or YJL130C)* New GPR: *YJR019C and YOR303W and YJL130C*	SL2ES *CPA2-URA2* is resolved correctly to 2 ESES for *CPA2* and *URA2* SL2ES *CPA1-URA2* is resolved correctly to one more ESES case *CPA1* At the same time it resolves 3 GES to ESES for the genes *CPA1*, *CPA2*, *URA2*	This captures the essentiality of all three genes consistent with literature.	[43] [44]
**Removal of reactions**	6	Remove orphan reaction r_2031 It was initially suggested in *i*AZ900	Reconciles GSL2 of *fur1-ura3* to SL2SL2	This removes an orphan reaction that might have added extra alternate paths to uridine formation	[8]
**Addition of GPR to orphan reactions**	7	Add genes for reaction r_0094 L-alanine [c] +pimeloyl-CoA [c] ⬄ 8-amino-7 oxononanoate [c] + CO_2_ [c] + CoA [c] + 4H^+^ [c]	Adds GPR: *YAR069W-A or YHR214W-F* Adds genes *BIO6* and *BIO8* putative genes to the model and both are correctly predicted as GG.	This makes the model better in terms of correct identification of non-essential genes.	[45]
	8	Add genes for reaction r_0475 H_2_O [c] + L-glutamine [c] ⬄ ammonium [c] + L-glutamate [c]	Adds GPR: *YMR096W or (YMR095C and YMR096W)* Adds genes *SNZ1* and *SNO1* to the model Correctly identifies *SNZ1* and *SNO1* genes as GG	This makes the model better in terms of correct identification of non-essential genes.	[46]

**Table 3:** Clarifications on 14 *in silico in vivo* inconsistencies.

No.	Gene	Inconsistency	Remarks	Reference
**1**	*SEC53*	ESG	*SEC53* deletion is *in silico* and *in vivo* essential, but it was erroneously categorized as non-essential.	[24] [47]
**2**	*HIS4*	ESG	*HIS4* gene deletion is lysine auxotroph, which is in corroboration with *in silico* result. Yet the *in vivo* strain was categorized as viable hence causing ESG inconsistency.	[48]
**3**	*ADK1*	ESG	*ADK1* gene *in vivo* deletion is not inviable initially but over a period of 4 days, cells fail to survive. *ADK1* *in silico* is adenine auxotroph as corroborated *in vivo.*	[49]
**4**	*ERG20*	ESG	*ERG20* deletion is *in silico* and *in vivo* essential, but it was erroneously categorized as non-essential.	[24] [50]
**5**	*URA4*	ESG	*URA4* gene deletion is uracil auxotroph and cell cycle is arrested by 87% over a period of 1 day. *In siico* simulations also reveal uracil auxotrophy but the *in vivo* strain was categorized as viable hence causing ESG inconsistency.	[51] [52] [53]
**6**	*MET2*	ESG	*MET2* gene deletion is methionine auxotroph and vegetative growth is reduced to less than 10%, which is in corroboration with *in silico* result. Yet, the *in vivo* strain was categorized as viable hence causing ESG inconsistency.	[51] [53]
**7**	*LYS2*	ESG	*LYS2* gene deletion is lysine auxotroph, which is in corroboration with *in silico* result. Yet, the *in vivo* strain was categorized as viable hence causing ESG inconsistency.	[54] [53]
**8**	*DPS1*	ESG	*DPS1* gene deletion is aspartate auxotroph, which is in corroboration with *in silico* result. Yet, the *in vivo* strain was categorized as viable hence causing ESG inconsistency.	[55]
**9**	*FRS1*	ESG	*FRS1* gene deletion is phenylalanine auxotroph, which is in corroboration with *in silico* result. Yet, the *in vivo* strain was categorized as viable hence causing ESG inconsistency.	[55]
**10**	*ADE13*	ESG	*ADE13* gene deletion is adenine auxotroph, which is in corroboration with *in silico* result. Yet, the *in vivo* strain was categorized as viable hence causing ESG inconsistency.	[54] [56] [57] [53]
**11**	*ADE4*	ESG	*ADE4* gene deletion is adenine auxotroph, which is in corroboration with *in silico* result. Yet, the *in vivo* strain was reported as viable hence causing ESG inconsistency.	[54] [56] [57] [53]
**12**	*RIB4*	ESG	*RIB4* gene deletion is riboflavin auxotroph, which is in corroboration with *in silico* result. Yet, the *in vivo* strain was categorized as viable hence causing ESG inconsistency.	[58] [53]
**13**	*TPI1*	GES	*TPI1* gene deletion is not *in silico* lethal. However, when PIT2m is suppressed, *TPI1* is essential for viability. This could possibly be because of short-term Crabtree effect due to F16-bisphosphate accumulation under *TPI1* deletion that suppresses mitochondrial respiratory enzymes.	[59] [60]
**14**	*FBA1*	GES	*FBA1* gene deletion is not *in silico* lethal. However, when PIT2m is suppressed, *FBA1* is essential for viability. This could possibly be because of short-term Crabtree effect due to F16-bisphosphate accumulation under *FBA1* deletion that suppresses mitochondrial respiratory enzymes.	[59] [60]

**Table 4:** Information about the 12 ESG cases, which form *in vivo* SL2 due to non-metabolic functions.

No.	*in vivo* Lethal Associations from Literature	Reason/Explanation	Reference
**1**	*RIB7* gene forms 2 lethal pairs: *RIB7-MAD1, RIB7-SGS1*	The candidate genes of lethal combination are non-metabolic and are involved in chromatid cohesion.	[57]
**2**	*HIS7* gene forms 1 lethal pair: *HIS7-RSP5*	*RSP5* is involved in endocytosis signaling pathway, a non-metabolic function, hence unable to be captured in a metabolic model.	[61]
**3**	*RIB5* gene forms 3 lethal pairs: *RIB5-BUB1, RIB5-MAD1, RIB5-TAF1*	The candidate genes of lethal combination are non-metabolic and are involved in mitosis.	[57]
**4**	*TSC10* gene forms 5 lethal pairs: *TSC10-CDC74*, *TSC10-CHL1, TSC10-MAD1, TSC10-MRE11, TSC10-SGS1*	The candidate genes of lethal combination are non-metabolic and are involved in chromatid cohesion.	[57]
**5**	*HEM13* gene forms 2 lethal pairs: *HEM13-CDC73, HEM13-SMC3*	The candidate genes of lethal combination are non-metabolic and are involved in chromatid cohesion.	[57]
**6**	*PRO3* gene forms 3 lethal pairs and 1 lethal triplet: *PRO3-CDC73, PRO3-LRP1, PRO3-NIP7, PRO3-GAP1-PUT4*	The candidate genes are non-metabolic in function.	[54] [57] [62]
**7**	*GNA1* forms 1 lethal pair: *GNA1-CHL1*	The lethality is owing to chromosome loss which is a non-metabolic phenomenon.	[57]
**8**	*FRS2* gene forms 5 lethal pairs: *FRS2-CDC73, FRS2-ELG1, FRS2-RAD51, FRS2-SGS1, FRS2-SMC3*	The candidate genes of lethal combination are non-metabolic and are involved in chromatid cohesion.	[57]
**9**	*TYS1* gene forms 2 lethal pairs: *TYS1-BUB1, TYS1-SGS1*	The candidate genes of lethal combination are non-metabolic and are involved in mitosis.	[57]
**10**	*ARG7* gene forms 1 lethal quadruplet: *ARG7-ALP1-CAN1-GAP1*	The quadruplet association is not entirely metabolic hence cannot be captured by metabolic model.	[54]
**11**	*OLE1* gene forms 3 lethal pairs: *OLE1-BUB1, OLE1-ELO1, OLE1-RML2*	*BUB1* gene is involved in mitosis. Δ*ole1*Δ*elo1* double mutant is inviable only in C:14 media *RML2* is non-metabolic gene	[57] [63] [64]
**12**	*YAH1* gene forms 1 lethal pair: *YAH1-MRE11*	*YAH1* has already been resolved as ESES *MRE11-YAH1* double knockout strain will result in meiotic recombination disorder and will be lethal. This is a non-metabolic attribute of yeast.	[57]

**Figure 2:**
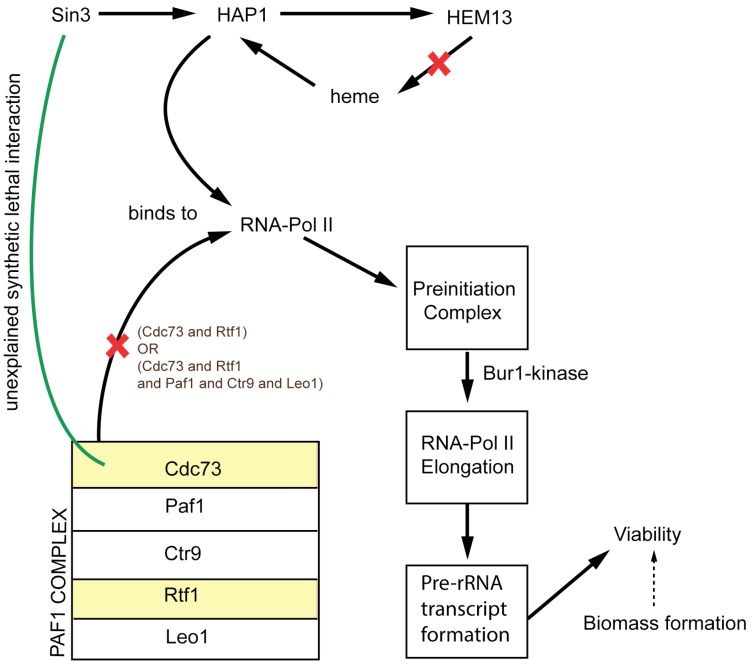
Shows a suggested schematic to exhibit the non-metabolic lethal interaction between *Cdc73* and *Hem13* gene in yeast. The red crosses represent the loss of function upon deletion of *Hem13* and *Cdc73* genes.

Overall, we reconciled 50 growth discrepancies (for full list of model modifications and comparison of the performance of *i*Sce926 and Yeast 7.11 see Supplemental File S1) between model and experiment (see Table 2 and Table 3). Twelve ESG cases were identified that form ESSL2 inconsistencies in combination with other non-metabolic genes (see Table 4). For example, gene *HEM13* whose deletion causes an ESG discrepancy has a non-metabolic function in chromatin assembly and interacts with RNA-polymerase II in transcription. It forms a synthetic lethal with *CDC73* [57] (cell division cycle gene) due to the inability to form the pre-rRNA transcript upon simultaneous deletion of the two. We propose a possible interaction schematic (see Figure 2) explaining the cause for the lethal interaction based on information from [65,66,67]. However, it is in general beyond the purview of a metabolic model to resolve inconsistencies whenever non-metabolic genes are implicated in the interaction.

Five separate classes of model modifications were introduced for Yeast 7.11 (see Table 2) including (a) addition of reactions, (b) removal of reactions, (c) GPR modifications, and (d) addition of GPR information for orphan reactions. We have also separately listed 12 ESG cases (see Table 3) where we have explained why they should have been ESES cases instead. 

#### 2.1.1. Addition of Reactions

A total of six reactions were added to Yeast 7.11 (see Supplemental File S1: Table 1). They generally fill in gaps in existing pathways by introducing *in vivo* verified reactions and the corresponding genes in the model. They also reconcile ESSL2 inconsistencies to SL2SL2 agreements. For example, restoring the *BAT1-BAT2* *in silico* synthetic lethal pair by adding α-keto isovalerate transport between mitochondria and cytosol in the model (see Table 2). Another reconciliation of ESG case to GG case involved an iron (II) transporter reaction being added to the model (see Table 2). Yeast 7.11 contains a low-affinity iron (II) transporter gene *FET4* causing ESG inconsistency. *In vivo* evidence [58] revealed the presence of a high-affinity iron (II) transporter (encoded by *FET3*) that can also transport iron (II) across the plasma membrane rendering the *FET3-FET4* gene combination an *in vivo* synthetic lethal. Thus, adding the *FET3* mediated transport reaction for iron (II) reconciles *FET4* from ESG to GG and also identifies *FET3-FET4* as an SL2SL2 match.

#### 2.1.2. Removal of Reactions

Three reactions (see Supplemental File S1: Table 1) were removed from the model that restored GSL2 inconsistencies to SL2SL2 agreements without affecting any of the correct predictions. For example, removal of the orphan reaction pyrimidine-nucleoside phosphorylase that converts uridine to UMP renders the *in silico* double mutant Δ*FUR1*Δ*URA3* lethal in agreement with *in vivo* data. The fact that the removed pyrimidine-nucleoside phosphorylase reaction lacked a GPR association possibly alludes to the putative nature of its inclusion into the model. The reactions (see Supplemental File S1: Table 1) were tagged using the IDs in the Yeast 7.11 model. One of suggested removals (*i.e.*, r_1682 where S-adenosyl L-methionine and zymosterol is converted to ergosta-5, 7, 22, 24 (28)-tetraen-3beta-ol) involved the elimination of a lumped form of a reaction whose elementary steps (*i.e.*, r_0986, r_0242, r_0243 and r_0244) are already present in the Yeast 7.11 model.

#### 2.1.3. GPR Modifications

**Figure 3:**
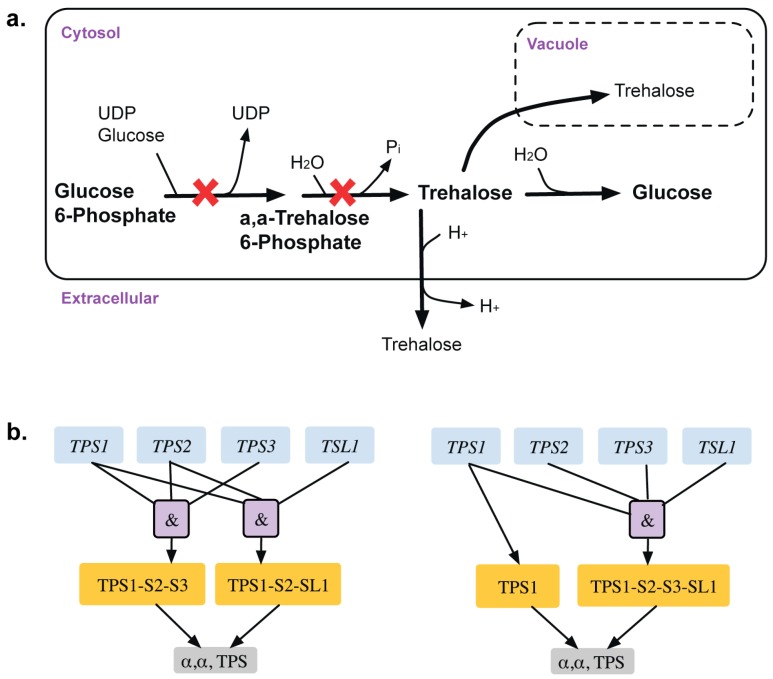
Trehalose metabolism. (**a**). Trehalose biosynthetic pathway with essential reactions marked with a red cross. (**b**). GPR modification revealing that *TPS1* gene is only essential for the associated reactions in minimal glucose medium. Old GPR is shown on the left and new GPR on the right.

A total of 13 GPR modifications were made to the model that corrected for 15 inconsistencies. For example, α, α-trehalose phosphatase synthase (UDP-forming) and trehalose phosphate phosphatase catalyze the formation of α,α-trehalose phosphate from glucose-6-phosphate, which is converted to trehalose in the presence of UDP-glucose (see Figure 3). The GPR amendment from (TPS1 and TPS2 and TPS3) or (TPS1 and TPS2 and TSL1) to TPS1 or (TPS1 and TPS2 and TPS3 and TSL1) changes the deletion of TPS2 from ESG to GG and identifies only TPS1 as essential [38,39] in glucose or fructose media as elucidated in Bell *et al.* [40].

In *S. cerevisiae*, most of the ATP formation occurs from glycolysis. A Δ*fba1* strain shows more than five-fold reduction in net ATP production *in silico,* which proportionately reduces the biomass flux (from 2.44 h^−1^ to 0.465 h^−1^). However, this is above the *in silico* viability threshold rendering the *fba* knockout non-essential. A possible reason for this contradiction could be the inability to capture the *in vivo* suppression of mitochondrial respiratory enzymes under accumulation of cytosolic fructose 1,6-bisphosphate due to short-term Crabtree effect [59]. In fact turning off *MIR1* mediated phosphate/proton mitochondrial symporter (PIt2m) [60] makes *fba1* mutant *in silico* lethal (reducing the net ATP production by ~35-fold) thereby resolving a GES case to an ESES case. The same observation also holds true for a *tpi1* deletion in *S. cerevisiae*.

**Figure 4:**
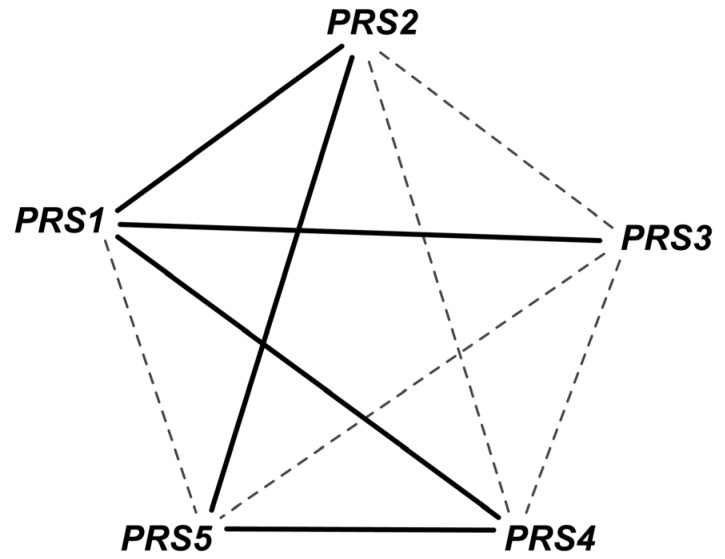
Gene combinations for PRPPS reaction. The gene pairs connected by solid edges are capable for catalyzing the reaction. Any such simultaneous gene deletion (such as *prs1-prs5* double deletion) that prevents the formation of all of these solid-line-connected gene pairs is lethal for the cell.

Phosphoribosyl diphosphate synthase catalyzes the essential reaction PRPPS. The inconsistency of this GPR was addressed in *i*AZ900 [8] and was modified to depict that any of the three PRS gene pairs (*PRS1* and *PRS3*), (*PRS2* and *PRS5*) or (*PRS4* and *PRS5*) is capable of encoding the sub-units required for catalyzing the reaction. However, it was later shown [68] that any of the five viable pairs (see Figure 4) need to be present for growth with one subunit containing an NHR (non-homologous region) and the other without one. Both *PRS1* and *PRS5* contain NHR while the rest do not. Therefore, the current GPR *(YHL011C and YKL181W) or (YOL061W and YBL068W) or (YOL061W and YER099C)* was corrected accordingly to *(YKL181W and YER099C) or (YKL181W and YHL011C) or (YKL181W and YBL068W) or (YER099C and YOL061W) or (YBL068W and YOL068W).* This correction not only recapitulates all *in vivo* observations but also predicts one lethal pair and two lethal triplet mutants (*i.e.*, Δ*prs1*Δ*prs5* and Δ*prs1*Δ*prs2*Δ*prs4*, Δ*prs2*Δ*prs3*Δ*prs4* respectively*).*

**Figure 5:**
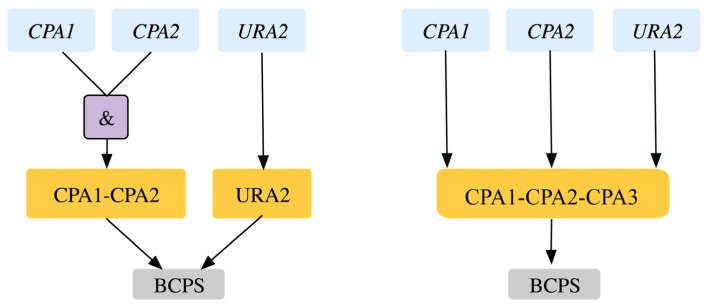
Gene-protein-reaction association for bifunctional carbamoyl phosphate synthase reaction. The genes (blue) code for the proteins (orange) that catalyze the reaction (gray). Old GPR is shown on the left and new GPR on the right.

**Figure 6:**
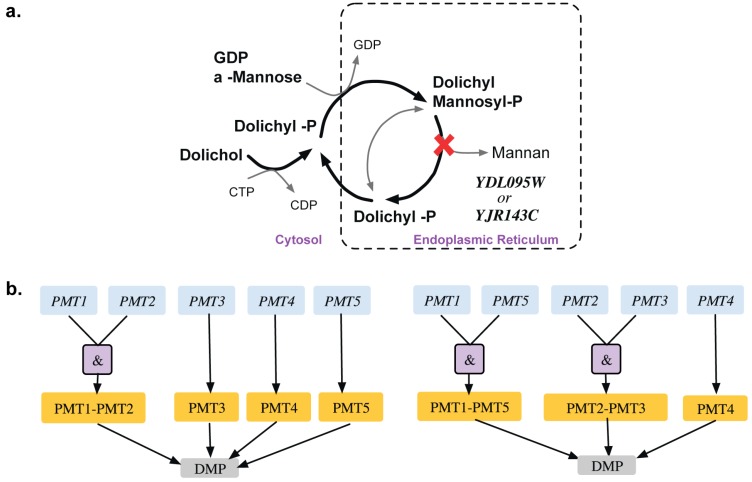
Auxotrophy of mannan (**a**). Pathway showing essential reaction (marked with red cross) for mannan biosynthesis (**b**). Gene-protein-reaction association for conversion of dolichyl-mannosyl phosphate to mannan. The genes (blue) code for the proteins (orange), which catalyze the reaction (gray). Old GPR is shown on the left and new GPR on the right.

Bifunctional carbamoyl phosphate synthetase catalyzes the first two enzymatic steps in the *de novo* biosynthesis of pyrimidines both of which undergo feedback inhibition by UTP (uridine tri-phosphate). The existing GPR identifies (*CPA1, URA2*) and (*CPA2, URA2*) as SL pairs. In fact, experimental evidence [43] and the Saccharomyces Genome Database [24] reveal that all three of *CPA1, CPA2 and URA2* are essential (see Figure 5). Similar evidence was seen in another yeast strain *Candida albicans* [44]. This GPR modification from *((YJR019C and YOR303W) or YJL130C))* to *(YJR019C and YOR303W and YJL130C)* rectifies two SL2ES cases to ESES and three GES cases to ESES. Dolichyl phosphate mannose mannosyl transferase catalyzes the conversion of dolichyl mannosyl phosphate to mannan in the endoplasmic reticulum, which is then transported out to the cytosol and forms a biomass precursor. The existing GPR (see Figure 6) suggests that either one of *PMT3*, *PMT4*, *PMT5* or both of *PMT1* and *PMT2* need to be present for the reaction to occur. However, recent experimental evidence [69,70] implies that PMT genes are classified in the following three sub-families (sub-family 1: *PMT1 and PMT5*), (sub-family 2: *PMT2 and PMT3*) and (sub-family 3: *PMT4*). Literature evidence [68] suggests that only the removal of *PMT4* in combination with deletions in both sub-family members 1 and 2 is lethal. Therefore, the GPR is modified from *((PMT1 and PMT2) or PMT3 or PMT4 or PMT5)* to *((PMT1 and PMT5) or (PMT2 and PMT3) or PMT4).* This modification puts forth the following four lethal triplet gene deletions: Δ*pmt1*Δ*pmt2*Δ*pmt4,* Δ*pmt5*Δ*pmt2*Δ*pmt4,* Δ*pmt1*Δ*pmt3*Δ*pmt4* and Δ*pmt5*Δ*pmt3*Δ*pmt4.*

#### 2.1.4. Addition of GPR to Orphan Reactions

There are 15 instances where GPR associations were assigned to orphan reactions in the model (see Supplemental File S1: Table 1). This adds 10 new gene loci to the model and correctly identifies them as non-essential based on growth data in budding yeast [43,44] and other well-annotated organisms such as *E. coli* [35]. For example, it has been shown [35] that *S. cerevisiae* contains an *E. coli* ortholog of *ubiC* gene that encodes for chorismate-pyruvate lyase enzyme, which enables the addition of the corresponding GPR to the reaction that was already present in the model. Gene deletion studies on *ubiC* reveal that it is non-essential matching model predictions. In another example, assigning *BIO6* and *BIO8* genes in the GPR for putative KAPA synthetase as seen in the YJM627 and A364a strains of *S. cerevisiae* [53,54] correctly identifies the deletion of either *BIO6* or *BIO8* as non-essential.

#### 2.1.5. MSL2 Gaps in the Model

**Figure 7:**
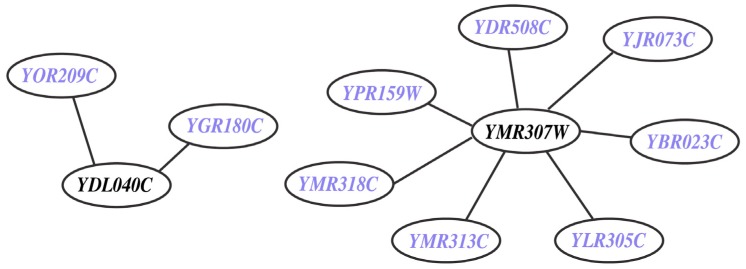
*In vivo* lethal gene pairs absent in model (MSL2 case). The genes *YDL040C* and *YMR307W* are involved in nine *in vivo* SL pairs but they are not present in Yeast 7.11 reconstruction. The participating genes (in blue) are found to be non-essential *in silico*.

This refers to missing (M) genes (*i.e.,* *YDL040C* and *YMR307W*) from the model despite the presence of experimental data on their deletion phenotype. They are integral to nine *in vivo* SL2 cases (see Figure 7 and Supplemental File S5). *YMR307W (GAS1)* encodes for beta-1,3-glucanosyltransferase [71] (belonging to the ERAD pathway [72]) a eukaryotic membrane protein embedded in the lipid bilayer that aids anchoring inositol associated glycophospholipids to the cell wall. On the other hand, *YDL040C (NAT1)* encodes for N-acetyltransferase and is primarily involved in cell wall integrity (CWI-MAPK) signaling pathway with a few ancillary functions such as cell-cycle, heat shock resistance, sporulation and telomeric silencing [73]. The CWI-MAPK signaling pathway [74,75]) involves a cohort of five genes *(i.e., NAT1, NAT2, NAT3, NAT4* and *NAT5*) that are missing in Yeast 7.11. In the current effort we have successfully incorporated *NAT1* and *NAT2* and have correctly identified them as GG cases.

### 2.2. Model Predictions for Synthetic Lethals in S. Cerevisiae

Experimental gene deletion compilations such as the Keio collection [15] and Saccharomyces Genome Database [22,44] are available for single gene deletion mutants. Exhaustive information for growth deficiency for single gene deletions is available but for higher gene deletion combinations the task becomes prohibitive. For example, *S. cerevisiae* genome with 3,912 metabolic genes would require approximately 7.5 million double-knockout experiments to verify the viability of all double deletion mutants. Computational tools, such as the SL finder can identify *in silico* synthetic lethal combinations thus narrowing down the combinations to be tested *in vivo*. Supplementary Tables 3 and 4 (see Supplemental File S4) tabulate the list of gene pairs and higher order gene deletions in Yeast 7.11 that are *in silico* lethal but have not been tested yet *in vivo*. While the lethal effect of some of these deletions is straightforward, a number of cases reveal non-intuitive lethal deletion combinations from distal parts of metabolism that either hinder cofactor synthesis or transport to another compartment. Four such cases identified using the corrected Yeast 7.11 model are described next in more detail.

#### 2.2.1. Proline Auxotrophy (Δ*pro1*Δ*car2* Double Mutant)

**Figure 8:**
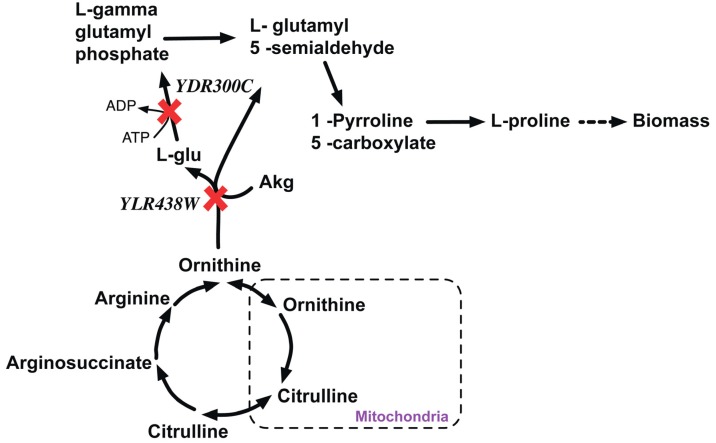
Proline metabolism disrupted due lethal gene pair knockout. *YDR300C* and *YLR438W* are genes encoding the proteins to catalyze the alternate paths for the formation of L-Gamma glutamyl phosphate that synthesizes in L-Proline. Red crosses mark knocked out genes.

*YDR300C (PRO1)* is a gamma-gluatmyl kinase that initiates proline biosynthesis by catalyzing the conversion of cytosolic glutamate to L-gamma glutamyl-5-phosphate. *In silico* removal of *PRO1* redirects the flux through the L-ornithine transaminase reaction to produce proline via L-glutamate 5-semialdehyde (LG5S) (see Figure 8). *In silico* removal of *YLR438W (*Δ*CAR2)* cannot catalyze the conversion of ornithine to LG5S. Therefore, the double deletion Δ*pro1*Δ*car1* strain cannot form LG5S and hence it is proline auxotrophic and thereby lethal.

#### 2.2.2. Leucine Auxotrophy (Δ*leu4*Δ*leu9* Double Mutant)

Δ*leu4*Δ*leu9* double mutant is devoid of α-isopropylmalate synthase (IPMS) activity leading to leucine auxotrophy. *YNL104C (LEU4)* encodes IPMS that catalyzes 2- isopropylmalate formation from 3-methyl 2-oxobutanoate essential in leucine biosynthesis. *YOR108W (LEU9)* is alpha-isopropylmalate synthase II and can carry out a residual α-IPMS activity in a **Δ***LEU4* strain. Existing *in vivo* studies [76] suggest the single deletions of *LEU4* or *LEU9* are non-essential which makes the double mutant a candidate for a synthetic lethal pair as suggested by the metabolic model.

#### 2.2.3. Arginine and Valine Auxotrophy (Δ*ctp1*Δ*mae1* Double Mutant)

**Figure 9:**
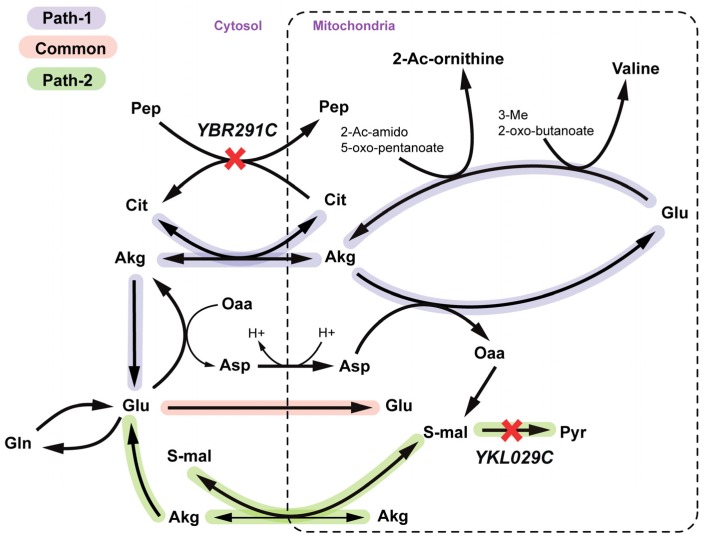
Valine and Arginine auxotrophy due to simultaneous deletion of *YBR291C* and *YKL029C* gene. Paths 1 and 2 represent two alternate routes to regenerate mitochondrial glutamate and the yellow path shows the part that is common to both paths. Mitochondrial valine goes to valine biosynthesis and 2-acetyl ornithine goes to arginine production. SL gene pairs are marked with red crosses.

*YBR291C (CTP1)* encodes the citrate-Pep antiporter from peroxisome and mitochondria to cytosol. *YKL029C (MAE1)* codes for the mitochondrial malic enzyme, which catalyzes oxidative decarboxylation of cytosolic S-malate to pyruvate (see Figure 9). There exist two alternate ways of providing mitochondrial L-glutamate (see Figure 9) required for valine and arginine biosynthesis. Pathway 1 uses Akg-citrate antiport to translocate mitochondrial Akg to cytosol reversibly, which is coupled to *ctp1* catalyzed citrate-pep antiport. Pathway 2 uses S-malate-Akg antiport to synthesize mitochondrial Akg which is prevented in a Δ*mae1* strain. A Δ*ctp1*Δ*mae1* double mutant is thus both cytosolic and mitochondrial-Akg auxotrophic and thereby is unable to synthesize mitochondrial L-glutamate.

#### 2.2.4. Disruption of Lipid Metabolism (Δ*itr1*Δ*ino1*Δ*itr2* triple mutant)

**Figure 10:**
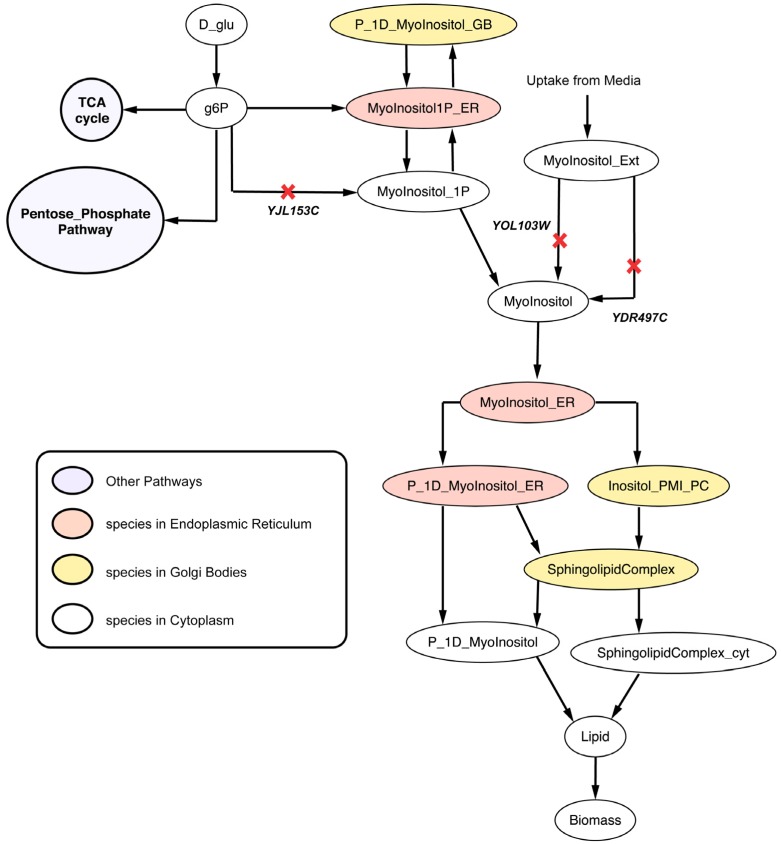
Lipid metabolism disrupted due deletion of synthetic lethal gene triplet. *YDR497C* and *YOL103W* are isozymes coding for the protein catalyzing the myo-inositol transport into the cytosol from media. Reactions catalyzed by SL gene triplet are marked with red crosses.

Lipid (a biomass precursor) is an intermediate metabolite formed by 15 precursor molecules in appropriate biological ratios in Yeast 7.11. The *in silico* Δ*YDR497C*Δ*YOL103W* (Δ*itr1*Δ*itr2*) double deletion strain cannot uptake myo-inositol thereby showing decrease in vegetative growth in corroboration with *in vivo* studies [77]. Δ*YJL153C* (Δ*ino1*) cannot convert cytosolic glucose-6-phosphate to myo-inositol-1-phosphate conversion according to the model (see Figure 10). Therefore, the Δ*itr1*Δ*ino1*Δ*itr2* triple mutant is found to be unable to form cytosolic myo-inositol and thus lipids. Single gene deletion studies [78,79] with inositol supplied in the media suggest that Δ*ino1* is not lethal but minimal media [23] without inositol makes *ino1* essential for viability. Note that *in silico* minimal media are supplemented with inositol (see Methods) in all calculations explaining why Δ*ino1* is found to be non-essential *in silico*.

The essentiality and synthetic analysis for the well-curated Yeast 7.11 model revealed a number of opportunities for model improvement and predictions for non-intuitive synthetic lethals to be tested experimentally for providing additional backing for the model and/or ways to remedy shortcomings. Subsequently, we switch our attention to a CHO cell model with significantly fewer mutant growth phenotypes tested.

### 2.3. C. Griseus Model CHO 1.2 Curation and Suggestion of Gene Knockout Experiments

The absence of a comprehensive single-gene knockout database for Chinese Hamster Ovary (CHO) cells (unlike yeast) makes the assessment CHO 1.2 genome-scale model [80] more difficult. Therefore, we supplemented limited experimental data with predicted lethal gene deletions based on the most recent mouse model [20] and gene knockout studies in mouse embryonic stem cells [81] that exhibited high degree of sequence similarity (functionality of the encoded protein is at least 70% conserved across all mammalian systems [81]) with the CHO cell genome. Any inconsistencies between mouse and CHO cell lethality was used as an opportunity to correct the CHO model (see Supplemental File S6). Eight GPR modifications were proposed for CHO 1.2 in order to address and reconcile five ESG cases to GG, three GES cases to ESES, three SL2ES cases to ESES and one ESSL2 case to SL2SL2. In addition, we propose a number of gene deletion experiments to verify non-intuitive synthetic lethal gene combinations. Reaction level essentiality analysis *in silico* revealed 90 essential reactions (see Table 5). Utilizing the GPR associations for these reactions, 57 essential genes were identified (see Supplemental File S4: Table 5) for growth under aerobic minimal essential media (media information in Supplemental File S3). A comparative analysis with existing experimental data, model modifications and suggested gene-deletion experiments have been listed in Table 6 (for full version of Table 6 see Supplemental File S1: Table 3).

**Table 5:** Chinese Hamster Ovary (CHO) 1.2 model statistics on ES and SL.

	Parameter	Count
Essentiality information	Essential Reactions with GPRs	82
	Essential Reactions without GPRs	8
	Essential genes	57
Reaction level lethality	SL Pairs	92
	SL Triplets	57
	SL Quadruplets	3
Gene level lethality	SL Pairs	43
	SL Triplets	20
	SL Quadruplets	3

**Table 6:** CHO 1.2 model essentiality and lethality comparisons with *in vivo* data and suggested gene deletion experiments.

		Gene Name	Comments	Modifications	Reference
**Single Gene Deletion**	***in silico* matches *in vivo***	*ggypS1*	Δ*ggypS1* mouse embyonic stem cells are embryonic lethal *in vivo. In silico* mutant strain cannot synthesize cholesterol, hence inviable. Thus *in vivo* result matches *in silico* predictions.		[82] [83]
	**GPR modifications to reconcile mismatch**	*gys1*	Δ*gys1* cannot produce glycogen *in silico*. However, *in vivo* studies show *gys1*- mutant is viable and forms SL2 with *gys2.*	GPR modified from: *(gys1 and gys2)* to *(gys1 or gys2)* *gys1* and *gys2* reconciled from ESG to GG *gys1-gys2* reconciles from ESG to SL2SL2	[84]
		*acsL1, acsL3, acsL4*	Δ*acsL4 in silico* mutant is sphingomyelin auxotroph. However, *in vivo* data for mouse reveals that *acsL4* deletion is viable.	r_0147 and r_0148 GPR was modified from *acsL4* to *(acsL1 or acsL3 or acsL4)* r_0142 GPR was modified from *acsL1* to *(acsL1 or acsL3 or acsL4)* r_0146 GPR was modified from *acsL3 to (acsL1 or acsL3 or acsL4)* *acsL1, acsL3* and *acsL4* were fixed from ESG to GG	[85]
	**Suggested experiment**	*qprT*	Δ*qprT* mutant *in silico* causes auxotrophy of cofactors NAD^+^, NADH, NADP^+^ and NADPH. No experimental evidence of knockout data exists in CHO-K1 cell line. This serves as a potential non-intuitive essential gene.		NA
**Double Gene Deletion**	**GPR modifications to reconcile mismatch**	*pgm1-pgm2*	Δ*pgm1*Δ*pgm2* double mutant is lethal *in silico* causing glycogen auxotrophy. However, single gene mouse deletion shows Δ*pgm2* strain is inviable and there is more than 80% homology in mouse and CHO *pgm2.* Thierry-Mieg *et al.* shows that *pgm2* is the major *PGM* isoform and is catalogued as MGI:97565.	GPR modification from *pgm1 or pgm2* to *pgm2 or (pgm1 and pgm2)*. *pgm2* is fixed from GES to ESESSL2ES case is fixed to ESES	[86] [87]
		*pcyT1a-pcyT1b*	Δ*pcyT1a*Δ*pcyT1b* double mutant causes phosphatidylcholine and sphingomyelin auxotrophy *in silico*. However, *in vivo* studies reveal that *pcyT1a* deletion alone is seen to be lethal in mouse.	Changing GPR for phoshphate cytidyltransferase reaction (r_1023) from *pcyT1a or pcyT1b* to *pcyT1a or (pcyT1a and pcyT1b)* resolves SL2ES to ESES and GES to ESES with respect to *pcyT1a.*	[88]
**Double Gene Deletion**	**GPR modifications to reconcile mismatch**	*chkA-cThkB*	Δ*chkA* mouse strains have been shown to be embryonic lethal. However Δ*chkB* deletions have been non-lethal.	Changing GPR for choline-kinase reactions r_0359 and r_0360 from *chkA or chkB* to *chkA or (chkA and chkB)* resolved SL2ES to ESES and GES to ESES with respect to *chkA*	[89]
	**Suggested experiment**	*slc14a1-slc14a2*	Δ*slc14a1*Δ*slc14a2* prevents spermidine and putrescine synthesis *in silico.* But there are no experimental evidence so it goes as a suggestion.		NA

#### 2.3.1. Suggested GPR Modifications to Reconcile Model Inconsistencies

**Figure 11:**
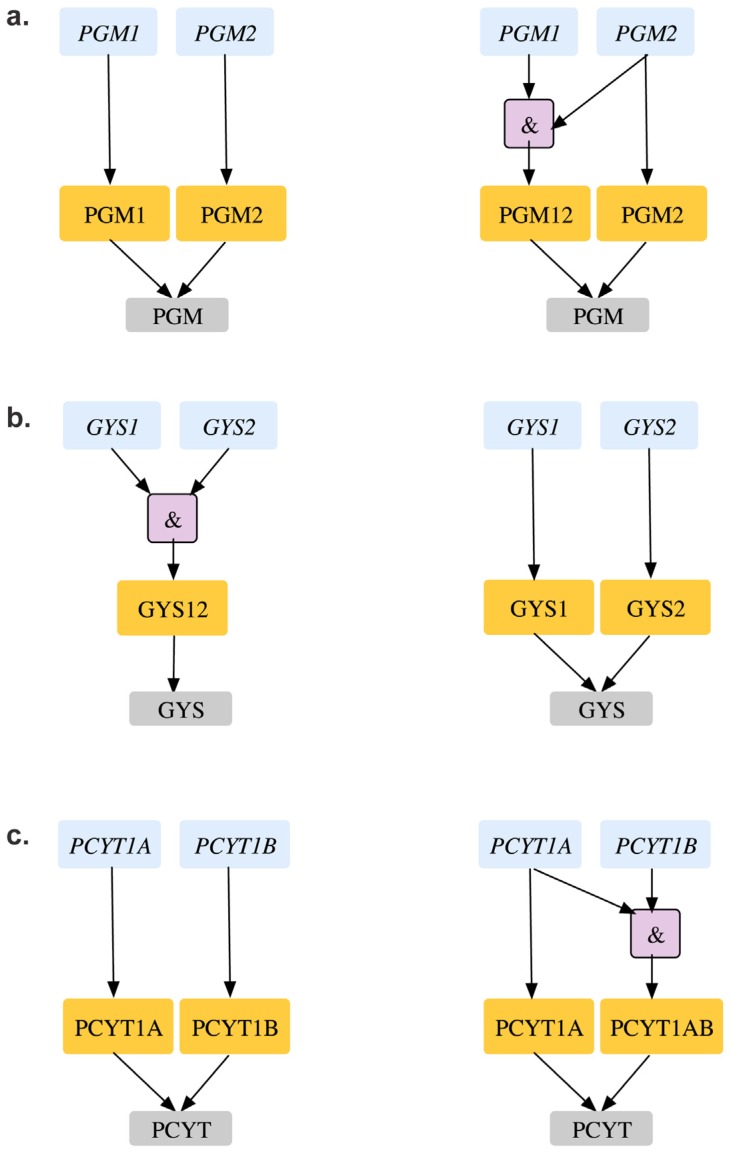
GPR modifications in CHO 1.2 model. (**a**). Reconciling *gys1* and *gys2* from essential genes to an SL2 (**b**). Shows how *pgm2* can be identified as an essential gene (**c**). Shows how *pcyT1a* can be identified as an essential gene. Old GPR is shown on the left and new GPR on the right.

Table 3 of Supplemental File S1 shows all eight GPR modifications in the CHO 1.2 model are based on *in vivo* gene deletion experiments [90] in CHO-K1 cell lines and embryonic stem cells of mouse [81]. For example, removal of glycogen synthase (GYS) reaction is lethal *in silico* as it blocks the pathway for synthesizing biomass precursor glycogen. The existing GPR (*gys1 and gys2)* suggests that deletion of either of the genes encoding the protein would be lethal *in silico* at the gene level. However, experiments on CHO K1 cell lines show that single deletion of these genes are not lethal *in vivo*. Furthermore, *in vivo* deletion experiments in related organisms with a conserved glycogen synthase activity (such as *S. cerevisiae* [54]) show that Δ*gys1*Δ*gys2* double deletion is lethal. As a result, the existing GPR was changed from (*gys1 and gys2)* to (*gys1 or gys2)* to reconcile two ESG and two ESSL2 inconsistencies to GG and SL2SL2 respectively (see Figure 11(a)). Unlike the previous inconsistency, we find a contrary case for *pgm2* mutant. The existing CHO 1.2 model suggests that either of *pgm1* or *pgm2* can encode for the *in silico* essential phosphoglucomutase (PGM) reaction required to synthesize biomass precursor glycogen (see Figure 11(b)). However, single gene deletion experiments in mice embryonic stem cells [91] show that deletion of *pgm1* is non-lethal as an active *pgm2* can compensate for loss of functional activity of *pgm1*. Deletion of *pgm2,* on the other hand is lethal, thus indicating that *pgm2* is the major isoform primarily responsible for PGM activity. As a result, the current GPR for PGM was accordingly changed from *pgm1 or pgm2* to *pgm2 or (pgm1 and pgm2)* that reconciles not only GES for *pgm2* to ESES but also SL2ES for *pgm1-pgm2* to ESES.

Similar to the *pgm2* case deletion of *pcyT1a* results in a GES inconsistency. Either of *pcyT1a* or *pcyT1b* can encode for the *in silico* essential choline phosphate cytidylyltransferase (CPCT) reaction. Removal of CPCT blocks the production of phosphatidylcholine and sphingomyelin, which are biomass precursors, thus making the two genes a synthetic lethal pair. However, *in vivo* single gene deletion studies in embryonic stem cells [88] show that while Δ*pcyT1a* is lethal, Δ*pcyT1b* mutant strains are viable. This observation suggests that *pcyT1a* is sufficient to encode for CPCT activity, while *pcyT1b* is a minor isoform. The GPR for CPCT was modified accordingly to *pcyT1a or (pcyT1a and pcyT1b)* to resolve *pcyT1a* GES to ESES and *pcyT1a-pcyT1b* SL2ES to ESES (see Figure 11c).

#### 2.3.2. Suggested Single and Double Gene Deletion Experiments

**Figure 12:**
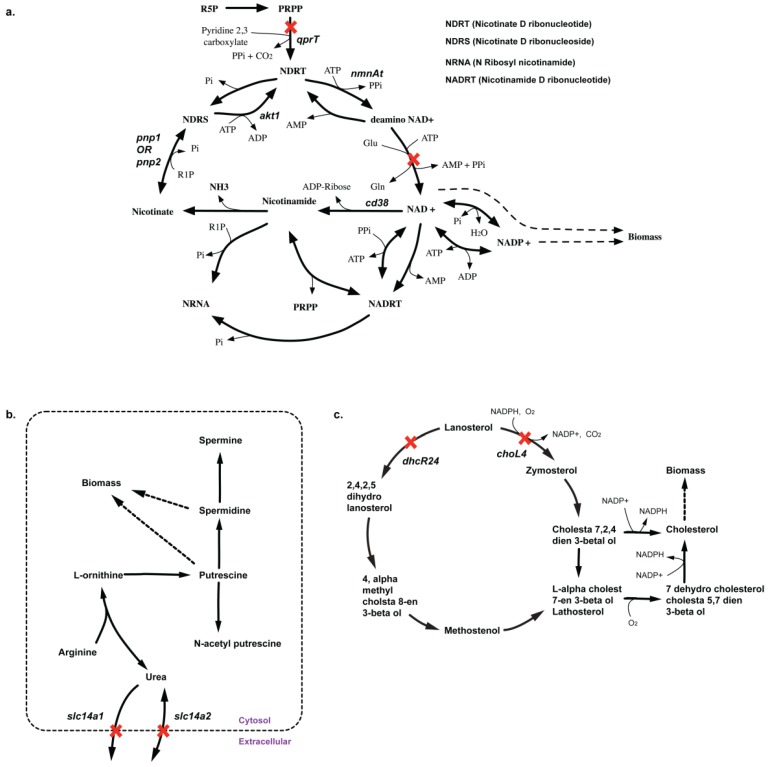
Pathways showing how biomass precursor(s) formation is blocked upon deletion of lethal gene(s). (**a**) Shows why *qprT* gene is essential for NAD-biosynthesis (**b**) Shows how deletion of *slc14a1* and *slc14a2* prevents synthesis of spermidine and putrescine (**c**) Shows how deletion of *dhcR24* and *choL4* prevents cholesterol biosynthesis.

Due to lack of sufficient experimental gene deletion data either in CHO or mouse embryonic stem cells, we have limited resources of confirming most of our *in silico* essential and synthetic lethal solutions. However, these identified sets could provide a blueprint for prioritizing future deletion experiments both for model curation as well as constructing high yielding phenotypes. In all, we identified 48 lethal reaction pairs and 44 SL gene pairs (see Supplemental File S4: Table 6) for CHO 1.2. Among these, we listed 10 non-intuitive combinations (see Supplemental File S1: Table 3) of gene deletions that can help improve the performance of the future CHO model reconstructions. For example, *in silico* deletion of *qprT*, encoding for quinolate phosphoribosyltransferase (QPRT) activity in NAD biosynthesis pathway, blocks the synthesis of biomass precursors NAD^+^, NADH, NADP^+^, and NADPH. Removal of QPRT prevents any fresh supply of nicotinate D-ribonucleotide (NDRT) to the nicotinamide regeneration cycle (see Figure 12(a)), thereby preventing any of the intermediate metabolite flux in the cycle (e.g., NAD^+^) to be diverted towards other pathways such as NADPH and biomass formation. The cycle functions as a futile cycle dissipating ATP. Production of all nicotinatmide-related cofactors (*i.e.*, NAD^+^, NADH, NADP^+^ and NADPH) are impaired resulting in zero biomass formation. Note that deletion of any of the genes (or gene pairs) encoding for the reactions in the nicotinamide regeration cycle (*i.e.*, *kynU, nmnAt* and *nadS*) are *in silico* essential (*pnp1* and *pnp2* form a lethal pair) for directly impairing activity of the cycle.

Another suggestion involves synthetic lethality caused by *in silico* removal of *slc14a1* and *slc14a2* genes. These genes encode for transporter proteins for urea whose removal prevents the export of urea from cytosol out of the cell. As there are no other pathways in the current CHO model to consume urea, deletion of urea export reactions prevents the activity of the urea cycle where it is synthesized. As a result, the model cannot synthesize intermediate metabolite ornithine required for the production of biomass precursor putrescine (see Figure 12(b)). The deletion of these genes can confirm if urea production is coupled with biomass or there are additional pathways of urea metabolism (or alternate pathways for ornithine and putrescine production) missing in the current reconstruction.

#### 2.3.3. SL2U Case Δ*dhcR24*Δ*choL4*

Similarly, *in silico* removal of both *dhcR24* and *choL4* cause complete loss of sterol delta-reductase (DSR) activity (see Figure 12(c)). Removal of DSR activity shuts off the cholesterol biosynthesis pathways *in silico*. Cholesterol is a biomass precursor, so DSR deletion *in silico* is causal to lethality. Deletion of *choL4* prevents zymosterol from being converted to dehydrocholesterol-cholesta 5, 7-dien 3-betaol (DC57D3B) (see Figure 12(c)) and ultimately to cholesterol in a cascade of four steps. However, cytosolic zymosterol can still be converted to *ebp*-encoded (emopamil binding protein- EBP) reaction forming N-cholesta 7, 2, 4-dien 3-betaol (NC724D3B) (see Figure 12(c)), which is subsequently converted to cholesterol. We note that EBP activity is common to both the routes (see Figure 12c), which makes *ebp* an essential gene for *in silico* cholesterol biosynthesis and subsequently biomass.

**Figure 13:**
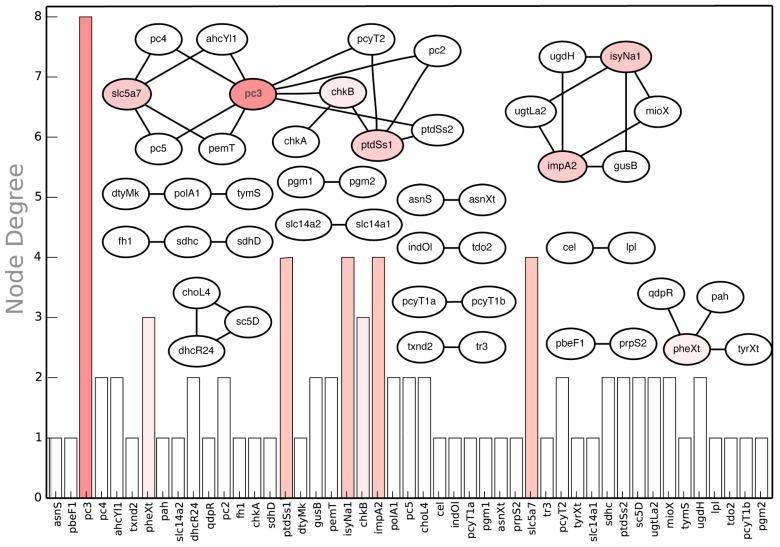
*In silico* SL2 landscape. It shows the synthetic lethal gene pair interactions present in the CHO 1.2 model along with the respective node degrees of each gene.

**Figure 14:**
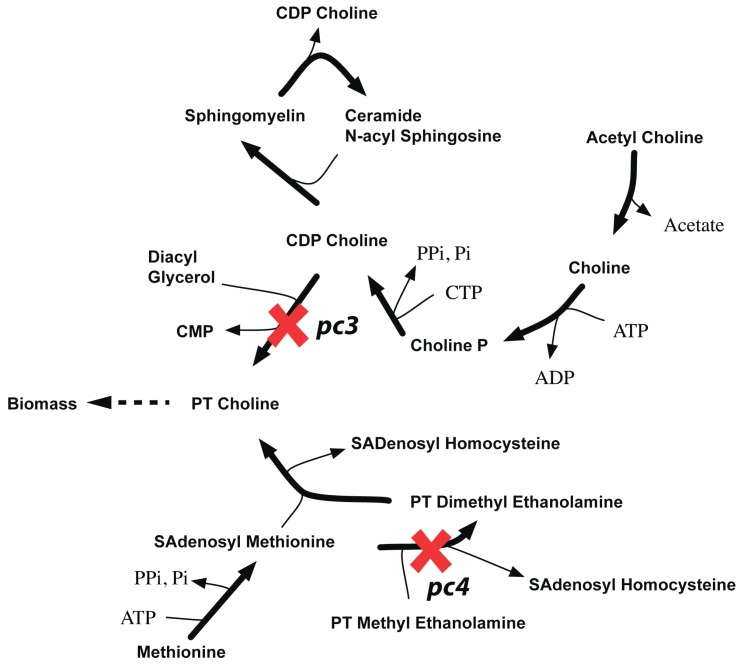
*pc3-pc4* synthetic lethality. It shows how *in silico* removal of *pc3* and *pc4* prevents synthesis of phosphatidyl choline.

In addition to manual inspection of *in silico* synthetic lethal suggestions for non-intuitive examples, we performed a node centrality analysis synthetic lethal landscape for CHO 1.2 (see Figure 13). Node centrality analysis is an important tool of querying complex networks (such as gene-association networks) to identify key nodes that have the maximal influence on the topology of the network [92]. In our case, we constructed the network of synthetic lethal gene pairs (see Figure 13) and ranked the genes based on the number of lethal pairs they were associated with. The graph shows that *pc3,* encoding for diacylglycerol choline phosphotransferase (DCP) activity in phosphatidyl choline (PTC) pathway, is associated with a maximum number of eight synthetic lethal gene pairs (see Figure 13). For example, *pc3* forms a lethal pair with *pc4* encoding for methylene-fatty-acyl-phospholipid synthase (MPS) reaction. *In silico* simultaneous gene deletion of *pc3* and *pc4* prevents synthesis of biomass precursor PTC synthesis (see Figure 14). Similarly, *impA2* is associated with four *in silico* SL2 (See Figure 13). One such case is lethality due to deletion of *impA2* and *ugtLa2* genes, which prevents the synthesis of biomass precursor phosphatidyl myoinositol. Note that the results of node centrality analysis (see Supplemental File S4: Table 8) can be utilized to prioritize the construction of mutant strains*.* For example, while Δ*pc3* strain can be utilized to verify *in silico-in vivo* consistency for eight synthetic lethal pairs, Δ*pbeF1* strain can be used to verify just a single case (*pbeF1-prpS2)*.

#### 2.3.4. Suggested Experiments for Higher Order Gene Deletions

We have further cataloged higher order lethal gene lists that show 20 lethal gene triplets and three lethal gene quadruplets. This is supplemented with information about the biomass precursor(s) each lethal mutant fails to synthesize (see Supplemental File S4: Tables 5–7). We hereby elucidate two higher order gene deletion experiments (see Table 6) to be tested in mouse embryonic stem cells or CHO-K1 cell lines based on the homology of gene functionality between mouse and CHO.

#### 2.3.5. SL3U Case Δ*cox(N)*Δ*sdhD*Δ*dhoDh*

**Figure 15:**
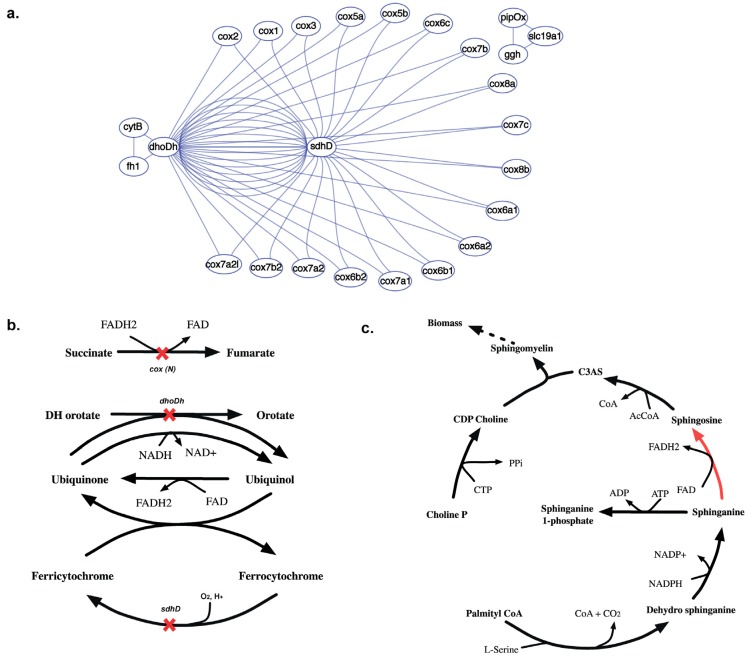
Synthetic lethal interaction of cytochrome oxidase genes. (**a**) Shows the SL3 landscape with *in silico* SL3 interactions between *sdhD, dhoDh* and 18 different *cox* genes. (**b**) Shows the reactions that get knocked out upon *in silico* removal of *dhoDh, sdhD* and either of the 18 *cox(N)* genes, preventing synthesis of cofactors FAD and FADH_2_. (**c**) Shows why the absence of FAD prevents the sphinganine to sphingosine conversion (shown in red) making the removal of gene triplet lethal.

The complex GPR relationship for the cytochrome-C oxidase (CCO) reaction contributes to 18 *in silico* SL3 combinations (see Figure 15(a)). Unlike any other reaction in this model, the CCO activity is performed by a holoenzyme that is encoded by 20 different *cox* genes. 18 of these 20 *cox* genes constitute lethal gene triplets with a putative succinate dehydrogenase (*sdhD*) and a dihydroorotate dehydrogenase (*dhoDh*). *In silico* removal of *cox(N)*, *sdhD* and *dhoDh* genes results in inability to regenerate cytosolic FAD (see Figure 15(b)). Consequently, FAD dependent sphinganine to sphingosine conversion is blocked that inhibits the synthesis of Ceramide 3-acyl sphingosine (C3AS). C3AS is required for the synthesis of biomass precursor sphingomyelin, which is blocked upon deletion of all the three genes thereby causing lethality.

#### 2.3.6. SL4U Case Δ*npl*Δ*nanS*Δ*st8Sia1*Δ*st8Sia5*

**Figure 16:**
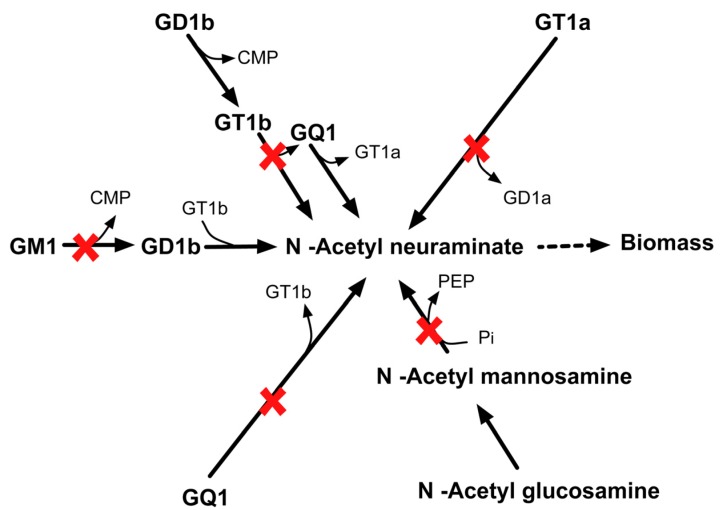
Lethality upon *in silico* removal of *npl, nanS*, *st8Sia1*, and *st8Sia5.* The quadruple mutant is unable to synthesize biomass precursor N-acetyl neuraminate.

Likewise, *in silico* quadruple deletion of *npl*, *nanS*, *st8Sia1* and *st8Sia5* genes inhibit anapyruvate lyase (APL) and sialyltransferase (SIL) activities. CHO 1.2 contains four different pathways of synthesizing N–acetyl neuraminate. Loss of all APL and SIL activities shut off all four pathways that form biomass precursor N–acetyl neuraminate (see Figure 16) *in silico* and thus leads to lethality.

## 3. Methods

The Synthetic Lethality Finder protocol [93] was applied for (i) the Yeast 7.11 [7] genome-scale model of *S. cerevisiae* under aerobic condition in minimal media and (ii) for the CHO 1.2 [80] model under aerobic conditions and minimal essential media [94] to identify essential reactions (for double, triple and higher order) deletions. The SL finder [93] identifies reaction (or combination thereof) in the metabolic network whose removal would restrict the maximum biomass below a pre-specified cutoff. This is solved using a min-max mixed-integer linear problem (MILP) where the inner problem maximizes biomass subject to network stoichiometry and nutrient uptake constraints. The outer problem, at the same time, identifies reaction whose removal would minimize the cellular biomass below a specified cut-off. By iteratively increasing the number of reaction deletion *k*, one can identify lethal reaction combinations of higher-order. For example, setting *k* equal to one identifies all essential reactions. Accordingly, *k = 2, 3…* refer to synthetic lethal pairs, triplets, etc. Upon identification of lethal reaction combinations, we elucidated the lethal deletion combinations at the gene level by making use of GPR associations. For example, in yeast, the essential holoenzyme acetyl-CoA carboxylase involves two functional subunits encoded by genes *ACC1* and *BPL1*. Deleting either of these genes prevents conversion of acetyl-CoA to malonyl-CoA therefore both genes are essential. In contrast, methylenetetrahydrofolate reductase is an essential reaction encoded by isozymes *MET13* and *MET12*. Both these genes need to be knocked-out to prevent the formation of 5-methyltetrahydrofolate from 5, 10-methylenetetrahydrofolate. Thus, in this case the reaction is essential but the associated genes form a synthetic lethal pair.

The minimal medium for yeast comprised of potassium, sodium, iron (II), nitrogen (as ammonia), sulfur (as sulfate) and phosphorus (as inorganic phosphate). Glucose was used as the sole carbon source. Trace nutrients such as 4-aminobenzoate, biotin, myo-inositol, nicotinate, pantothenic acid and thiamin were included [95]*.* Glucose and oxygen uptake rates were set at 100 and 20 mmol gDW^−1^h^−1^, respectively (in accordance with the experimental study [96] which showed that oxygen uptake is about a fifth of glucose uptake on a molar basis for aerobic growth in yeast). The non-growth associated ATP maintenance was set at 1 mmol gDW^−1^h^−1^ as proposed by Mo *et al.* [95]. Maximum biomass (FBA) calculations for CHO cells were carried out under minimal essential media [80] (used for CHO cell culture as shown in Riordan *et al.* [97]*,* Stanners *et al.* [98]) with glucose, amino acids (Val, Lys, Leu, Thr, Met, Arg and His) and all four nucleotides along with nitrogen sources. 10% of the maximum theoretical biomass was chosen for both cases as the cutoff for growth [99]. We used the mixed integer program CPLEX solver in GAMS using an Intel Xeon X5675 Six-Core 3.06 GHz with 48GB of physical memory, for reaction level results and Python v3.4.1 for all calculations.

The effect of gene deletions on biomass component production was also systematically assessed using a Precursor Identifier algorithm (see Supplemental File S2). Once a lethal gene deletion combination is identified, the corresponding biomass component whose production is compromised is identified along with the inactivated production pathway. Python scripts were used to generate topology files for most networks drawn in this paper in *DOT* graphing language. Graphs were displayed using Omnigraffle 6.0^TM^ operating on the python scripts and post-processed. The updated *S. cerevisiae* model was generated using COBRA Toolbox v2.0 [100] in Excel and SMBL formats respectively (see Supplemental Files S7 and S8).

## 4. Conclusions

In this study we propose 50 model modifications for Yeast 7.11 and eight modifications for CHO 1.2 that improve their model consistencies in terms of essential and lethal gene predictions. In addition, we have also suggested non-intuitive gene deletion combinations for both yeast and CHO-K1 cell lines for experimental validation that can aid in future curation of the genome-scale models. Overall, the contribution of multi-gene deletion data to enhance the performance of genome-scale metabolic reconstructions has been demonstrated in this work. In addition, our analysis identifies several cases where the metabolism-only description of the current models fails to reconcile *in silico-in vivo* inconsistencies (ESG cases in Yeast) arising due to non-metabolic interactions. Incorporation of detailed information for gene transcription and translation in genome-scale models, as described in the ME-model formalisms (for *E. coli* [101] and *T. maratima* [102]), offer opportunities for reconciling these inconsistencies. In case of CHO 1.2 model, however, the major obstacle towards model curation remains to be the paucity of gene deletion information for CHO-K1 cell lines, or the related mouse embryonic stem cells. A comprehensive gene deletion databank, similar to that available for *E. coli* an *S. cerevisiae*, will greatly contribute towards improving the performance of the current model.

## Supplementary Files

Supplementary File 1

## Appendix

Description of the Abbreviations

**GPR—**Gene Protein Reaction association

**ES—**Essential genes

**G—**Genes whose singular deletion does not affect cell viability

**SLx—**Set of x genes whose simultaneous deletion is lethal. For example, SL3 refers to a set of 3 genes that constitute a lethal triplet

**ESES—**Genes whose deletion reduces cellular growth below the viability threshold for both *in silico* and *in vivo* cases

**GG—**Genes which when deleted *in silico* and knocked out *in vivo* does not decrease cell growth in both cases

**GES—**This represents the list of genes which when deleted *in silico* does not affect cell growth. However, *in vivo* knockout of such genes, leads to cell death.

**SL2ES—**A non-essential gene whose simultaneous deletion with another non-essential gene is lethal *in silico*, however, at least one of which is essential *in vivo*.

**ESSL2—**This are those gene pairs for which a simultaneous knockout of both genes *in vivo* causes cell death. However, at least one of the genes of the pair when knocked out *in silico* causes cell death.

**SL2G—**This is the list of gene pairs where simultaneous deletion of both genes *in silico* causes cell death. However, a simultaneous *in vivo* knockout of both genes is non-lethal for the cell.

**GSL2—**This is the list of gene pairs where simultaneous deletion of both genes is non-lethal *in silico*. However, a simultaneous *in vivo* knockout of both genes is lethal for the cell.

**SL2SL2—**Simultaneous removal of the genes of this list of gene pairs causes cell death *in silico* and *in vivo* both. However, a single gene deletion was not lethal in both *in silico* and *in vivo.*

**SL2U—**Simultaneous deletion of the genes of this list of gene pairs causes *in silico* lethality. However, there exist no *in vivo* evidence for the same. This puts them to the *in vivo* untested (**U**) category.

**Specificity, Sensitivity and False Viable Rate calculations:**

The specificity of a model is defined as follows:

**Specificity = #ESES/(#ESES + #GES)** [8]

Using this metric, the selectivity of Yeast 7.11 is:

${\mathrm{Specificity}}_{Yeast7.11}\;=\;\frac{\#ESES}{\#ESES+\#GES}=\frac{72}{72+178}=0.288$

In contrast, the specificity of our current model *i*Sce926 is:

${\mathrm{Specificity}}_{iSce926}\;=\;\frac{\#ESES}{\#ESES+\#GES}=\frac{92}{92+173}=0.347$, which shows a 20.4% improvement.

Likewise, the sensitivity (or True Viable Rate) of a model is defined as follows:

**Sensitivity = #GG/(#GG + #ESG) [8]**

Using this metric, the sensitivity of Yeast 7.11 is:

${\mathrm{Sensitivity}}_{Yeast7.11}\;=\;\frac{\#GG}{\#GG+\#ESG}=\frac{583}{583+30}=0.951$

In contrast, the sensitivity of our current model *i*Sce926 is:

${\mathrm{Sensitivity}}_{iSce926}\;=\;\frac{\#GG}{\#GG+\#ESG}=\frac{603}{603+14}=0.977$, which shows a 2.66% improvement.

Again, the False Viable Rate (**FVR**) is defined as:

**FVR = #GES/(#GES+#ESES) [8]**

Using this metric, the FVR of Yeast 7.11 is:

${\mathrm{FVR}}_{Yeast7.11}\;=\;\frac{\#GES}{\#GES+\#ESES}=\frac{178}{178+72}=0.712$

In contrast, the FVR of our current model *i*Sce926 is:

${\mathrm{FVR}}_{iSce926}\;=\;\frac{\#GES}{\#GES+\#ESES}=\frac{173}{173+92}=0.652$, which shows a reduction by 8.42%.
