# Supplementary material for: Using Gene Essentiality and Synthetic Lethality Information to Correct Yeast and CHO Cell Genome-Scale Models
Source: Metabolites. 2015 Sep 29;5(4):536–70. doi: 10.3390/metabo5040536 (PMC4693185; doi:10.3390/metabo5040536)
Supplement: Supplementary File 1 [file metabolites-05-00536-s001.zip › S1.pdf]

Supplemental File 1:
Table 1. Full version of Table 2 showing comprehensive list of model modifications

	No.	Model Modification	Improvement on Yeast 7.11	Reference
Addition of Reactions to Yeast 7.11	1	Addition of alpha-keto isovalerate transport<br>3-methyl 2-oxobutanoate [m] ⇔ 3-methyl 2-oxobutanoate [**c**]	• *BAT1* reconciled from ESG to GG<br>• *BAT1-BAT2* reconciled from SL2ES to SL2SL2.	[1]<br>[2]
	2	Addition of pretyrosine formation<br>prephenate [c] + L-glutamate [c] ⇔ 2-oxoglutarate [c] + pretyrosine [c]<br>GPR: *YGL202W and YHR137W*	Addition of prephenate aminotransferase in the pretyrosine pathway and also retains *YGL202W* and *YHR137W* as GG.	[3]<br>[4]<br>[5]
	3	Addition of pretyrosine dehydratase<br>pretyrosine [c] ⇔ CO_2 [c] + H_2O [c] + L-phenylalanine [c]<br>GPR: *YNL316C*	Addition of pretyrosine dehydratase in the pretyrosine pathway also retaining *YNL316C* as GG.	[6]
	4	Addition of tyrosine degradation pathway<br>3-(4-hydroxyphenyl) pyruvate [c] -> CO_2 [c] + 3-(4-hyroxyphenyl) acetaldehyde [c]<br>GPR: unknown EC 4.1.1.80	Addition of (4-hydroxyphenyl) pyruvate decarboxylase in the tyrosine degradation pathway. The ORF for the gene is unknown but this pathway is non-essential for viability hence adds to GG.	[7]<br>[8]
	5	Addition of tyrosine degradation pathway<br>3-(4-hyroxyphenyl) acetaldehyde [c] + NADH [c] -><br>NAD^+ [c] + tyrosol [c]<br>GPR: unknown EC 1.1.1.1	Addition of reduction reaction to form alcohol from aldehyde in tyrosine degradation pathway is similarly non-essential for viability and so GG.	[7]<br>[8]
	6	Mitochondrial acetyl-transferase activity of glycine<br>CoA [c] + L-2 amino 3-oxobutanoate [c] ⇔ acetyl-CoA [m] + L-glycine [m]<br>GPR: *YDL040C or YGR147C or YHR013C*	• Correctly adds *NAT1, NAT2* and *ARD1* as GG cases.	[9]<br>[10]
	7	GPR modification for reaction r_0195<br>Old GPR: *((YBR126C and YDR074W and YMR261C) or (YML100W and YBR126C and YDR074W))*<br>New GPR: *((YBR126C and YDR074W and YMR261C and YML100W) or YBR126C)*	• *TPS1* gene is only essential in glucose media whereas both *TPS1* and *TPS2* genes are essential in galactose media reflected in old GPR<br>• *TPS2* gene is restored as a GG from an ESG	[11]<br>[12]<br>[13]
	8	GPR modification for reaction r_1051<br>Old GPR: *((YBR126C and YDR074W and YMR261C) or (YML100W and YBR126C and YDR074W))*<br>New GPR: *((YBR126C and YDR074W and YMR261C and YML100W) or YBR126C)*	• *TPS1* gene is only essential in glucose media whereas both *TPS1* and *TPS2* genes are essential in galactose media reflected in old GPR<br>• *TPS2* gene is restored as a GG from an ESG	[11]<br>[12]<br>[13]
	9	GPR modification for reaction r_0995<br>Old GPR: *YDR023W or YHR011W*	• *SES1* gene is corrected from GES to ESES<br>• *SES1-DIA1* is corrected from SL2ES and SL2G cases to	[14]<br>[15]

		New GPR: *YDR023W or (YDR023W and YHR011W)*	ESES and GG cases respectively.	
GPR modifications	10	GPR modification for reaction r_0916<br>Old GPR: *((YHL011C and YKL181W) or (YOL061W and YBL068W) or (YOL061W and YER099C))*<br>New GPR: *((YKL181W and YER099C) or (YKL181W and YHL011C) or (YKL181W and YBL068W) or (YER099C and YOL061W) or (YBL068W and YOL068W))*	• The new GPR suggests 4 new probable lethal triplet mutants namely: *Δprs1Δprs2Δprs4*, *Δprs1Δprs2Δprs5*, *Δprs1Δprs3Δprs5*, *Δprs2Δprs3Δprs4* and *Δprs1Δprs4Δprs5*	[16]
	11	GPR modification for reaction r_1178<br>Old GPR: *YMR319C*<br>New GPR: *YMR319C or YMR058W*	• Iron (II) transporter across plasma membrane has 2 different genes encoding under low-affinity and high-affinity conditions<br>• *FET4* gene is reconciled from ESG to GG<br>• *FET3* gene is added to the model as a non-essential gene<br>• *FET3-FET4 in vivo* lethal gene pair is correctly predicted by model now	[17]
	12	GPR modification for reaction r_0250<br>Old GPR: *((YJR019C and YOR303W) or YJL130C)*<br>New GPR: *YJR019C and YOR303W and YJL130C*	• SL2ES *CPA2-URA2* is resolved correctly to 2 ESES cases *CPA2* and *URA2*<br>• SL2ES *CPA1-URA2* is resolved correctly to one more ESES case *CPA1*<br>• At the same time it resolves 3 GES to ESES cases for the genes *CPA1*, *CPA2*, *URA2*	[18]<br>[19]
	13	GPR modification for reactions r_0886 and r_0887 along with suppression of PIT2m reaction (r_1245) due to Crabtree effect<br>Old GPR: *YMR205C and YGR240C*<br>New GPR: *YMR205C or (YGR240C and YMR205C)*	• Resolves *YDR050C-YMR205C* SL2ES case to ESES<br>• Resolves *YDR050C-YGR240C* SL2G cases to GG<br>• Resolves *YMR205C-YGR240C* GSL2 to SL2SL2<br>• Resolves *tpi1-fba1* SL2ES to ESES cases<br>• Resolves *tpi1* from GES to ESES<br>• Resolves *fba1* from GES to ESES	[20]<br>[21]
	14	GPR modification of reaction r_2116<br>Old GPR: *ALD2*<br>New GPR: *ALD2 or ALD5 or ALD4*	• Resolves *ALD2* from SL2G to GG	[22]
	15	GPR modification of reaction r_0020<br>Old GPR: *YDR035W*<br>New GPR: *YDR035W or YBR249C*	• Resolves *MAE1* from SL2G to GG	[23]
	17	GPR modification of reaction r_0116<br>Old GPR: *PHO11 or PHO5*<br>New GPR: *PHO11 or PHO12 or PHO3 or PHO5*	• Adds genes *PHO5 and PHO12* to the model and correctly identified as GG	[24]

	18	GPR modification of reaction r_1237<br>Old GPR: *YOR130C*<br>New GPR*: YOR130C or BAC1*	• Resolves *ORT1* from ESG to GG<br>• Prediction of SL2U for *ORT1-BAC1*	[25]
	19	GPR modification for reaction r_0005<br>Old GPR: *YGR032W or YLR342W*<br>New GPR: *(YGR032W or YLR342W) and (YCR034W or YLR372W)*	• Identifies *YCR034W-YLR372W* as SL2SL2 from GSL2 cases	[26]
	20	GPR modification for reaction r_0888<br>Old GPR: *YMR105C or YKL127W*<br>New GPR: *(YMR105C or YKL127W) and (YMR105C or YKL127W or YMR278W)*	• Retains *PGM1-PGM2* SL2SL2 case and adds *PGM3* as a GG in the model.	[27]
**Removal of reactions**	21	Remove orphan reaction r_2031<br>It was initially suggested in *i*AZ900	Reconciles GSL2 of *fur1-ura3* case to SL2SL2	
	22	Remove reaction r_1682	Removes duplicate reaction, which is a lumped version of reactions r_0986, r_0243, r_0242, r_0244.	[28]
	23	Remove reaction r_0461	• Should not be present in *S. cerevisiae*, seen in *M. mazei* and also confirmed in UniProt.	[29]
**Add genes to orphan reactions**	24	Add an *E. coli* like *ubiC* gene for reaction r_1685. We call it here *Y-ubiC* gene.<br>Chorismate [c] ⇔ 4-hydroxybenzoate [c] + pyruvate [c]<br>GPR: unknown	• The ORF is unknown but the gene has been identified as GG.<br>• Adds GPR to an orphan reaction	[30]<br>[31]<br>[32]
	25	Add genes for reaction r_0026<br>4-methyl thio-2-oxobutanoate [c] + L-glutamate [c] ⇔ 2-oxoglutarate [c] + L-methionine [c]	• Adds GPR: *YHR208W or YJR148W or YGL202W or YHR137W* where all 4 genes are correctly identified as GG	[33]
	26	Add genes for reaction r_0043<br>3-hexaprenyl-4-hydroxybenzoic acid [c] + 0.5 oxygen [c] ⇔ 3-hexaprenyl-4,5 dihydroxybenzoate [c]	Adds GPR: *YPL252C or YDR376W*<br>Both the genes are correctly identified as GG	[34]
	27	Add genes for reaction r_0044<br>3-hexaprenyl-4-hydroxy-5-methoxybezoic acid [m] + H^+ [m] ⇔ 2-hexaprenyl-6-methoxyphenol [m] + CO_2 [m]	Adds GPR: *YDR538W and YDR539W*<br>Both the genes are correctly identified as GG	[35]

28	Add genes for reaction r_0086<br>S-methyl-5-thio-D-ribulose 1-phosphate [c] ⇔ 5-methyl sulfanyl-2,3-dioxopentyl phosphate [c] + H_2O [c]	Adds GPR: *YJR024C*<br>It is correctly identified as GG	[36]
29	Add genes for reaction r_0087<br>S-methyl-5-thio alpha ribose 1-phosphate [c] ⇔ S-methyl-5-thio-D-ribulose 1-phosphate [c]	Adds GPR: *YPR118W*<br>It is correctly identified as GG	[33]
30	Add genes for reaction r_0094<br>L-alanine [c] +pimeloyl-CoA [c] ⇔ 8-amino-7 oxononanoate [c] + CO_2 [c] + CoA [c] + $4H^+$ [c]	• Adds GPR: *YAR069W-A or YHR214W-F*<br>• Adds genes *BIO6* and *BIO8* putative genes to the model and both are correctly predicted as GG.	[37]
31	Add genes for reaction r_0475<br>H_2O [c] + L-glutamine [c] ⇔ ammonium [c] + L-glutamate [c]	• Adds GPR: *YMR096W or (YMR095C and YMR096W)*<br>• Adds genes *SNZ1* and *SNO1* to the model<br>Correctly identifies *SNZ1* and *SNO1* genes as GG	[38]
32	Add genes for reaction r_0992<br>Acetyl-CoA [c] + L-serine [c] ⇔ CoA [c] + O-acetyl-L-serine [c]	• Adds GPR: *(YDL040C and YGR147C and YHR013C) or (YDL040C and YGR147C)*	[39]
33	Add genes for reaction r_1623<br>5-formyltetrahydro folate [m] + ATP [m] ⇔ 5,10 methenyl-THF [m] + ADP [m] + P_i [m]	Adds GPR: *YER183C*	[40]
34	Add genes for reaction r_1624<br>5-formyltetrahydro folate [c] + ATP [c] + H_2O [c] ⇔ 10-formyl THF [c] + ADP [c] + P_i [c] + H^+ [c]	Adds GPR: *YER183C*	[40]
35	Adds GPR to reaction r_1603<br>GPR: *ADE5,7*	Correctly identified to be a GG	[41]
36	Adds GPR to reaction r_0696<br>GPR: *YOL151W (GRE2)*	Correctly identified to be a GG	[42]
37	Adds GPR to reaction r_1739<br>GPR: *PAA1*	Correctly identified to be a GG	[43]
38	Adds GPR to reaction r_1790<br>GPR: *YDL045C*	Correctly identified to be a GG	[44]

Table 2. Summary of results showing improvement of ES and SL prediction of *i*Sce926 over Yeast 7.11

Description	*i*Sce926	Yeast 7.11	Comments
Correct prediction of *in vivo* essential genes = ESES	92	72	*i*Sce926 shows 27.7% better performance than Yeast 7.11
Correct prediction of *in vivo* synthetic lethal genes = SL2SL2+SL3SL3	17	23	*i*Sce926 shows 35.2% better performance than Yeast 7.11
Reduction of erroneous prediction of *in vivo* non-essential genes = ESG	30	14	*i*Sce926 shows 53.3% reduction in erroneous prediction than Yeast 7.11
Suggested lethal knockout experiments (pairs and triples) = SL2U+SL3U	15	27	*i*Sce926 shows 80% increase in suggested gene deletion experiments than Yeast 7.11
Selectivity= ESES/(ESES+GES)	0.347	0.288	*i*Sce926 shows 20.4% increase in selectivity than Yeast 7.11
Specificity= GG/(GG+ESG)	0.951	0.977	*i*Sce926 shows 2.7% increase in specificity than Yeast 7.11

Table 3. Full version of Table 6 showing agreement, disagreement with *in vivo* data and model modifications in CHO 1.2

		Gene name	Comments	Modifications	Reference
**Single Gene Deletion**	Match between *in vivo* and *in silico*	*acsL3*	Δ*acsL3* strain has an impaired Liver-X Receptor/ Retinoid-X Receptor signalling pathway in CHO cells. CHO-K1 cell lines show mutant strains have impaired lipid biosynthesis. *In silico* mutant strain shows sphingomyelin auxotrophy. However, *in vivo* lethality endorses *in silico* predictions.		[45]<br>[46]<br>[45]
		ggypS1	Δ*ggypS1* strain has an impaired carotenoid biosynthesis *in vivo.* Similarly, mouse and human Δ*ggypS1* cell lines were also found to be inviable since it is an important G-protein precursor. *In silico* mutant strain is cholesterol auxotroph, hence inviable. Thus *in vivo* result matches *in silico* predictions.		[47]<br>[48]<br>[47]
		fasN	*fasN* gene is shown to be highly conserved in mammalian systems. Δ*fasN* strain has been shown to shut off the lipid biosynthesis and hence renders the strain inviable in mouse. It can be extrapolated as a compliance of *in vivo* and *in silico* results.		[49]<br>[50]
		hmgCr	*hmgCr* gene shows more than 80% homology in CHO cells with humans and mouse counterparts. *In vivo* mouse and *in silico* CHO Δ*hmgCr* strains are cholesterol auxotrophs and thus affirm conformation.		[51]
	Mismatch between *in vivo* and *in silico*	*dhfr*	Deletion of *dhfr* prevents biomass precursor sphingomyelin formation *in silico*, However, this deletion is not lethal in vivo in CHO-K1 cell lines.		[46]
		gys1	Δ*gys1 in silico* mutant strain is glycogen auxotroph. *in vivo* studies show *gys1*- mutant is viable and forms SL2 with *gys2.*	GPR modified from: *(gys1 and gys2)* to *(gys1 or gys2)*<br>*gys1* and *gys2* reconciled from ESG to GG<br>*gys1-gys2* reconciles from ESG to SL2SL2	[52]<br>[53]<br>[54]
		acsL1, acsL3, acsL4	Δ*acsL4 in silico* mutant is sphingomyelin auxotroph. However, *in vivo* data for mouse reveals that *acsL4* deletion is viable.	r_0147 and r_0148 GPR was modified from *acsL4* to *(acsL1 or acsL3 or acsL4)*<br>r_0142 GPR was modified from *acsL1* to *(acsL1 or acsL3 or acsL4)*<br>r_0146 GPR was modified from *acsL3 to (acsL1 or acsL3 or acsL4)*<br>*acsL1, acsL3* and *acsL4* were fixed from ESG to GG	[55]
		afmld	Δ*afmld in silico* mutant is sphingomyelin auxotroph. However, *in vivo* data for mouse reveals that *afmld* deletion is viable.		[56]

**Single gene deletion**	Suggestion for CHO-K1 single gene deletion	*arg1*	Δ*arg1* mutant *in silico* is spermidine and putrescine auxotroph. Mouse knockout experiments show that it is *in vivo* essential. But due to lack of gene homology information, this serves as an important suggestion for an experimentalist to perform in CHO-K1 cell line.		[57]
		mthFr	Δ*mthFr* mutant *in silico* is 5-methyl tetrahydrofolate auxotroph and hence inviable. Mouse knockout experiments show that it is *in vivo* essential. Unavailability of homology information makes it an important suggestion for CHO-K1 single gene deletion candidate.		[58]
		qprT	Δ*qprT* mutant *in silico* causes auxotrophy of cofactors NAD^+, NADH, $NADP^+$ and NADPH. No experimental evidence of knockout data exists in CHO-K1 cell line. This serves as a potential non-intuitive essential gene.		NA[1]
		ugp2	Δ*ugp2* mutant *in silico* causes glycogen auxotrophy. No experimental evidence of CHO-K1 knockout data for *ugp2* exists. This makes it an important suggestion for single gene knockout studies.		NA
			.		
**Double gene deletion**	Mismatches with experimental evidence	*pgm1-pgm2*	Δ*pgm1*Δ*pgm2* double mutant is lethal *in silico* causing glycogen auxotrophy. However, single gene mouse deletion shows Δ*pgm2* strain is inviable and there is more than 80% homology in mouse and CHO *pgm2.*	GPR modification from *pgm1 or pgm2* to *pgm2 or (pgm1 and pgm2)*. *pgm2* is fixed from GES to ESES SL2ES case is fixed to ESES	[59]
		pcyT1a-pcyT1b	Δ*pcyT1a*Δ*pcyT1b* double mutant causes phosphatidylcholine and sphingomyelin auxotrophy *in silico*. However, *in vivo* studies reveal that *pcyT1a* deletion alone is seen to be lethal in mouse.	Changing GPR for phoshphate cytidyltransferase reaction (r_1023) from *pcyT1a or pcyT1b* to *pcyT1a or (pcyT1a and pcyT1b)* resolves SL2ES to ESES and GES to ESES with respect to *pcyT1a.*	[60]
		chkA-chkB	Δ*chkA* mouse strains have been shown to be embryonic lethal. However Δ*chkB* deletions have been non-lethal.	Changing GPR for choline-kinase reactions r_0359 and r_0360 from *chkA or chkB* to *chkA or (chkA and chkB)* resolved SL2ES to ESES and GES to ESES with respect to *chkA*	[61]
	Suggested CHO-K1 experiments	*slc14a1-slc14a2*	Δ*slc14a1*Δ*slc14a2* double mutant has been shown to be spermidine and putrescine auxotroph. In a mouse *in silico* reconstruction[2] this has been shown to be lethal pair as well. But there are no experimental evidence so it goes as a suggestion.		NA
		dhcR24-choL4	Δ*dhcR24*Δ*choL4* double mutant is cholesterol auxotroph in both CHO 1.2 and mouse *in silico* models. However no experimental single or double deletion data exists.		NA

	Suggested CHO-K1 experiments	*ptdSs1-ptdSs2*	Δ*ptdSs1*Δ*ptdSs2* is incapable of *in silico* production of phosphatidylserine and phosphatidylethanolamine. The mouse GSM also confirms this *in silico* lethal pair. However no experimental single or double deletion data exists in either CHO-K1 or mouse.	NA
		gusB-impA2	Δ*gusB*Δ*impA2* is incapable of *in silico* production of 1-phosphatidyl-D-myoinositol. The mouse GSM also confirms this *in silico* lethal pair. However no experimental single or double deletion data exists in either CHO-K1 or mouse.	NA
**Higher order gene deletions**	Suggested CHO-K1 experiments	*dhoDh-fh1-cytB*	Δ*dhoDh*Δ*fh1*Δ*cytB* triple mutants are incapable of Sphingomyelin production *in silico.* Neither *in silico* higher gene deletion information was available from the mouse model nor do we have any mouse or CHO *in vivo* triple knockout information.	NA
		ggh-pipOx-slc19a1	Δ*ggh*Δ*pipOx*Δ*slc19a1* triple mutants are incapable of 5 methyl tetrahydrofolate production *in silico.* Neither *in silico* higher gene deletion information was available from the mouse model nor do we have any mouse or CHO *in vivo* triple knockout information.	NA
		cox(N)-dhoDh-sdhD	Δ*cox(N)*Δ*dhoDh*Δ*sdhD* triple mutants are incapable of Sphingomyelin production *in silico.* Neither *in silico* higher gene deletion information was available from the mouse model nor do we have any mouse or CHO *in vivo* triple knockout information. N belongs to {1, 2, 3, 5a, 5b, 6a1, 6a2, 6b1, 6b2, 6c, 7a1, 7a2, 7a2l, 7b, 7c, 8a, 8b} This particular example points to 18 *in silico* lethal triplets, but we have explained them in results as a single case.	NA
		nanS-npl-st8Sia1 -st8Sia5	Δ*nanS*Δ*npl*Δ*st8Sia1*Δ*st8Sia5* quadruple mutants are incapable of N-acetylneuraminate production *in silico.* Neither *in silico* higher gene deletion information was available from the mouse model nor do we have any mouse or CHO *in vivo* quadruple knockout information.	NA
		ak1-ak2-ak3L1 -cmpK1	Δ*ak1*Δ*ak2*Δ*ak3L1*Δ*cmpK1* quadruple mutants are incapable of phosphatidylglycerol, phosphatidylserine, phosphatidylethanolamine, phosphatidylcholine, cardiolipin, 1-phosphatidyl-D-myoinositol production *in silico.* Neither *in silico* higher gene deletion information was available from the mouse model nor do we have any mouse or CHO *in vivo* quadruple knockout information.	NA

polA1-rrm1-rrm2 -rrm2b	Δ*polA1*Δ*rrm1*Δ*rrm2*Δ*rrm2b* quadruple mutants are incapable of d-ATP, d-TTP, d-CTP and d-GTP production *in silico.* Neither *in silico* higher gene deletion information was available from the mouse model nor do we have any mouse or CHO *in vivo* quadruple knockout information.	NA

1. Baudin, A.; Ozier-Kalogeropoulos, O.; Denouel, A.; Lacroute, F.; Cullin, C. A simple and efficient method for direct gene deletion in saccharomyces cerevisiae. *Nucleic acids research* **1993**, *21*, 3329-3330.
2. Harrison, R.; Papp, B.; Pal, C.; Oliver, S.G.; Delneri, D. Plasticity of genetic interactions in metabolic networks of yeast. *Proceedings of the National Academy of Sciences of the United States of America* **2007**, *104*, 2307-2312.
3. Kradolfer, P.; Niederberger, P.; Hutter, R. Tryptophan degradation in saccharomyces cerevisiae: Characterization of two aromatic aminotransferases. *Archives of microbiology* **1982**, *133*, 242-248.
4. Urrestarazu, A.; Vissers, S.; Iraqui, I.; Grenson, M. Phenylalanine- and tyrosine-auxotrophic mutants of saccharomyces cerevisiae impaired in transamination. *Molecular & general genetics : MGG* **1998**, *257*, 230-237.
5. Iraqui, I.; Vissers, S.; Cartiaux, M.; Urrestarazu, A. Characterisation of saccharomyces cerevisiae aro8 and aro9 genes encoding aromatic aminotransferases i and ii reveals a new aminotransferase subfamily. *Molecular & general genetics : MGG* **1998**, *257*, 238-248.
6. Maftahi, M.; Nicaud, J.M.; Levesque, H.; Gaillardin, C. Sequencing analysis of a 24.7 kb fragment of yeast chromosome xiv identifies six known genes, a new member of the hexose transporter family and ten new open reading frames. *Yeast* **1995**, *11*, 1077-1085.
7. Sentheshanuganathan, S. The mechanism of the formation of higher alcohols from amino acids by saccharomyces cerevisiae. *The Biochemical journal* **1960**, *74*, 568-576.
8. Dickinson, J.R. Pathways of leucine and valine catabolism in yeast. *Methods Enzymol* **2000**, *324*, 80-92.
9. Gollub, E.G.; Liu, K.P.; Dayan, J.; Adlersberg, M.; Sprinson, D.B. Yeast mutants deficient in heme biosynthesis and a heme mutant additionally blocked in cyclization of 2,3-oxidosqualene. *The Journal of biological chemistry* **1977**, *252*, 2846-2854.
10. Oh-hama, T. Evolutionary consideration on 5-aminolevulinate synthase in nature. *Origins of life and evolution of the biosphere : the journal of the International Society for the Study of the Origin of Life* **1997**, *27*, 405-412.
11. De Silva-Udawatta, M.N.; Cannon, J.F. Roles of trehalose phosphate synthase in yeast glycogen metabolism and sporulation. *Molecular microbiology* **2001**, *40*, 1345-1356.
12. de Lichtenberg, U.; Jensen, L.J.; Brunak, S.; Bork, P. Dynamic complex formation during the yeast cell cycle. *Science* **2005**, *307*, 724-727.
13. Bell, W.; Sun, W.; Hohmann, S.; Wera, S.; Reinders, A.; De Virgilio, C.; Wiemken, A.; Thevelein, J.M. Composition and functional analysis of the saccharomyces cerevisiae trehalose synthase complex. *The Journal of biological chemistry* **1998**, *273*, 33311-33319.
14. Barros, M.H.; Nobrega, F.G. Yah1 of saccharomyces cerevisiae: A new essential gene that codes for a protein homologous to human adrenodoxin. *Gene* **1999**, *233*, 197-203.
15. Barros, M.H.; Carlson, C.G.; Glerum, D.M.; Tzagoloff, A. Involvement of mitochondrial ferredoxin and cox15p in hydroxylation of heme o. *Febs Lett* **2001**, *492*, 133-138.
16. Hove-Jensen, B. Heterooligomeric phosphoribosyl diphosphate synthase of saccharomyces cerevisiae: Combinatorial expression of the five prs genes in escherichia coli. *The Journal of biological chemistry* **2004**, *279*, 40345-40350.

17. Berthelet, S.; Usher, J.; Shulist, K.; Hamza, A.; Maltez, N.; Johnston, A.; Fong, Y.; Harris, L.J.; Baetz, K. Functional genomics analysis of the saccharomyces cerevisiae iron responsive transcription factor aft1 reveals iron-independent functions. *Genetics* **2010**, *185*, 1111-1128.
18. Lim, A.L.; Powers-Lee, S.G. Requirement for the carboxyl-terminal domain of saccharomyces cerevisiae carbamoyl-phosphate synthetase. *The Journal of biological chemistry* **1996**, *271*, 11400-11409.
19. Inglis, D.O.; Arnaud, M.B.; Binkley, J.; Shah, P.; Skrzypek, M.S.; Wymore, F.; Binkley, G.; Miyasato, S.R.; Simison, M.; Sherlock, G. The candida genome database incorporates multiple candida species: Multispecies search and analysis tools with curated gene and protein information for candida albicans and candida glabrata. *Nucleic acids research* **2012**, *40*, D667-674.
20. Diaz-Ruiz, R.; Averet, N.; Araiza, D.; Pinson, B.; Uribe-Carvajal, S.; Devin, A.; Rigoulet, M. Mitochondrial oxidative phosphorylation is regulated by fructose 1,6-bisphosphate. A possible role in crabtree effect induction? *The Journal of biological chemistry* **2008**, *283*, 26948-26955.
21. Guaragnella, N.; Zdralevic, M.; Antonacci, L.; Passarella, S.; Marra, E.; Giannattasio, S. The role of mitochondria in yeast programmed cell death. *Frontiers in oncology* **2012**, *2*, 70.
22. Navarro-Avino, J.P.; Prasad, R.; Miralles, V.J.; Benito, R.M.; Serrano, R. A proposal for nomenclature of aldehyde dehydrogenases in saccharomyces cerevisiae and characterization of the stress-inducible ald2 and ald3 genes. *Yeast* **1999**, *15*, 829-842.
23. Tkach, J.M.; Yimit, A.; Lee, A.Y.; Riffle, M.; Costanzo, M.; Jaschob, D.; Hendry, J.A.; Ou, J.; Moffat, J.; Boone, C.*, et al.* Dissecting DNA damage response pathways by analysing protein localization and abundance changes during DNA replication stress. *Nature cell biology* **2012**, *14*, 966-976.
24. Shnyreva, M.G.; Petrova, E.V.; Egorov, S.N.; Hinnen, A. Biochemical properties and excretion behavior of repressible acid phosphatases with altered subunit composition. *Microbiological research* **1996**, *151*, 291-300.
25. Zomorrodi, A.R.; Maranas, C.D. Improving the imm904 s. Cerevisiae metabolic model using essentiality and synthetic lethality data. *BMC systems biology* **2010**, *4*, 178.
26. Oh, C.S.; Toke, D.A.; Mandala, S.; Martin, C.E. Elo2 and elo3, homologues of the saccharomyces cerevisiae elo1 gene, function in fatty acid elongation and are required for sphingolipid formation. *The Journal of biological chemistry* **1997**, *272*, 17376-17384.
27. Walther, T.; Baylac, A.; Alkim, C.; Vax, A.; Cordier, H.; Francois, J.M. The pgm3 gene encodes the major phosphoribomutase in the yeast saccharomyces cerevisiae. *Febs Lett* **2012**, *586*, 4114-4118.
28. Herrgard, M.J.; Swainston, N.; Dobson, P.; Dunn, W.B.; Arga, K.Y.; Arvas, M.; Bluthgen, N.; Borger, S.; Costenoble, R.; Heinemann, M.*, et al.* A consensus yeast metabolic network reconstruction obtained from a community approach to systems biology. *Nature biotechnology* **2008**, *26*, 1155-1160.
29. Ogawa, T.; Yoshimura, T.; Hemmi, H. Connected cavity structure enables prenyl elongation across the dimer interface in mutated geranylfarnesyl diphosphate synthase from methanosarcina mazei. *Biochemical and biophysical research communications* **2011**, *409*, 333-337.
30. Meganathan, R. Ubiquinone biosynthesis in microorganisms. *FEMS microbiology letters* **2001**, *203*, 131-139.
31. Nichols, B.P.; Green, J.M. Cloning and sequencing of escherichia coli ubic and purification of chorismate lyase. *Journal of bacteriology* **1992**, *174*, 5309-5316.
32. Siebert, M.; Severin, K.; Heide, L. Formation of 4-hydroxybenzoate in escherichia coli: Characterization of the ubic gene and its encoded enzyme chorismate pyruvate-lyase. *Microbiology* **1994**, *140 (Pt 4)*, 897-904.
33. Petti, A.A.; Crutchfield, C.A.; Rabinowitz, J.D.; Botstein, D. Survival of starving yeast is correlated with oxidative stress response and nonrespiratory mitochondrial function. *Proceedings of the National Academy of Sciences of the United States of America* **2011**, *108*, E1089-1098.
34. Ozeir, M.; Muhlenhoff, U.; Webert, H.; Lill, R.; Fontecave, M.; Pierrel, F. Coenzyme q biosynthesis: Coq6 is required for the c5-hydroxylation reaction and substrate analogs rescue coq6 deficiency. *Chemistry & biology* **2011**, *18*, 1134-1142.
35. Mukai, N.; Masaki, K.; Fujii, T.; Kawamukai, M.; Iefuji, H. Pad1 and fdc1 are essential for the decarboxylation of phenylacrylic acids in saccharomyces cerevisiae. *Journal of bioscience and bioengineering* **2010**, *109*, 564-569.
36. Pirkov, I.; Norbeck, J.; Gustafsson, L.; Albers, E. A complete inventory of all enzymes in the eukaryotic methionine salvage pathway. *The FEBS journal* **2008**, *275*, 4111-4120.
37. Hall, C.; Dietrich, F.S. The reacquisition of biotin prototrophy in saccharomyces cerevisiae involved horizontal gene transfer, gene duplication and gene clustering. *Genetics* **2007**, *177*, 2293-2307.

38. Tobias, S.; Rajic, I.; Vanyi, A. Effect of t-2 toxin on egg production and hatchability in laying hens. *Acta veterinaria Hungarica* **1992**, *40*, 47-54.
39. Gautschi, M.; Just, S.; Mun, A.; Ross, S.; Rucknagel, P.; Dubaquie, Y.; Ehrenhofer-Murray, A.; Rospert, S. The yeast n(alpha)-acetyltransferase nata is quantitatively anchored to the ribosome and interacts with nascent polypeptides. *Molecular and cellular biology* **2003**, *23*, 7403-7414.
40. Holmes, W.B.; Appling, D.R. Cloning and characterization of methenyltetrahydrofolate synthetase from saccharomyces cerevisiae. *The Journal of biological chemistry* **2002**, *277*, 20205-20213.
41. Henikoff, S. The saccharomyces cerevisiae ade5,7 protein is homologous to overlapping drosophila melanogaster gart polypeptides. *Journal of molecular biology* **1986**, *190*, 519-528.
42. Guo, P.C.; Bao, Z.Z.; Ma, X.X.; Xia, Q.; Li, W.F. Structural insights into the cofactor-assisted substrate recognition of yeast methylglyoxal/isovaleraldehyde reductase gre2. *Biochimica et biophysica acta* **2014**, *1844*, 1486-1492.
43. Liu, B.; Sutton, A.; Sternglanz, R. A yeast polyamine acetyltransferase. *The Journal of biological chemistry* **2005**, *280*, 16659-16664.
44. Wu, M.; Repetto, B.; Glerum, D.M.; Tzagoloff, A. Cloning and characterization of fad1, the structural gene for flavin adenine dinucleotide synthetase of saccharomyces cerevisiae. *Molecular and cellular biology* **1995**, *15*, 264-271.
45. Poppelreuther, M.; Rudolph, B.; Du, C.; Grossmann, R.; Becker, M.; Thiele, C.; Ehehalt, R.; Fullekrug, J. The n-terminal region of acyl-coa synthetase 3 is essential for both the localization on lipid droplets and the function in fatty acid uptake. *Journal of lipid research* **2012**, *53*, 888-900.
46. Dong, B.; Kan, C.F.; Singh, A.B.; Liu, J. High-fructose diet downregulates long-chain acyl-coa synthetase 3 expression in liver of hamsters via impairing lxr/rxr signaling pathway. *Journal of lipid research* **2013**, *54*, 1241-1254.
47. Ruppel, N.J.; Kropp, K.N.; Davis, P.A.; Martin, A.E.; Luesse, D.R.; Hangarter, R.P. Mutations in geranylgeranyl diphosphate synthase 1 affect chloroplast development in arabidopsis thaliana (brassicaceae). *American journal of botany* **2013**, *100*, 2074-2084.
48. Kainou, T.; Kawamura, K.; Tanaka, K.; Matsuda, H.; Kawamukai, M. Identification of the ggps1 genes encoding geranylgeranyl diphosphate synthases from mouse and human. *Biochimica et biophysica acta* **1999**, *1437*, 333-340.
49. Chirala, S.S.; Chang, H.; Matzuk, M.; Abu-Elheiga, L.; Mao, J.; Mahon, K.; Finegold, M.; Wakil, S.J. Fatty acid synthesis is essential in embryonic development: Fatty acid synthase null mutants and most of the heterozygotes die in utero. *Proceedings of the National Academy of Sciences of the United States of America* **2003**, *100*, 6358-6363.
50. Chakravarthy, M.V.; Pan, Z.; Zhu, Y.; Tordjman, K.; Schneider, J.G.; Coleman, T.; Turk, J.; Semenkovich, C.F. "New" hepatic fat activates pparalpha to maintain glucose, lipid, and cholesterol homeostasis. *Cell metabolism* **2005**, *1*, 309-322.
51. Ohashi, K.; Osuga, J.; Tozawa, R.; Kitamine, T.; Yagyu, H.; Sekiya, M.; Tomita, S.; Okazaki, H.; Tamura, Y.; Yahagi, N*., et al.* Early embryonic lethality caused by targeted disruption of the 3-hydroxy-3-methylglutaryl-coa reductase gene. *The Journal of biological chemistry* **2003**, *278*, 42936-42941.
52. Douillard-Guilloux, G.; Raben, N.; Takikita, S.; Ferry, A.; Vignaud, A.; Guillet-Deniau, I.; Favier, M.; Thurberg, B.L.; Roach, P.J.; Caillaud, C*., et al.* Restoration of muscle functionality by genetic suppression of glycogen synthesis in a murine model of pompe disease. *Human molecular genetics* **2010**, *19*, 684-696.
53. Castro, E.; Eeles, R. The role of brca1 and brca2 in prostate cancer. *Asian journal of andrology* **2012**, *14*, 409-414.
54. Dedes, K.J.; Wilkerson, P.M.; Wetterskog, D.; Weigelt, B.; Ashworth, A.; Reis-Filho, J.S. Synthetic lethality of parp inhibition in cancers lacking brca1 and brca2 mutations. *Cell cycle* **2011**, *10*, 1192-1199.
55. Cho, Y.Y.; Kang, M.J.; Sone, H.; Suzuki, T.; Abe, M.; Igarashi, M.; Tokunaga, T.; Ogawa, S.; Takei, Y.A.; Miyazawa, T*., et al.* Abnormal uterus with polycysts, accumulation of uterine prostaglandins, and reduced fertility in mice heterozygous for acyl-coa synthetase 4 deficiency. *Biochemical and biophysical research communications* **2001**, *284*, 993-997.
56. Dubrovsky, A.L.; Taratuto, A.L.; Sevlever, G.; Schultz, M.; Pegoraro, E.; Hoop, R.C.; Hoffman, E.P. Duchenne muscular dystrophy and myotonic dystrophy in the same patient. *American journal of medical genetics* **1995**, *55*, 342-348.
57. Iyer, R.K.; Yoo, P.K.; Kern, R.M.; Rozengurt, N.; Tsoa, R.; O'Brien, W.E.; Yu, H.; Grody, W.W.; Cederbaum, S.D. Mouse model for human arginase deficiency. *Molecular and cellular biology* **2002**, *22*, 4491-4498.

58. Chen, Z.; Karaplis, A.C.; Ackerman, S.L.; Pogribny, I.P.; Melnyk, S.; Lussier-Cacan, S.; Chen, M.F.; Pai, A.; John, S.W.; Smith, R.S., *et al.* Mice deficient in methylenetetrahydrofolate reductase exhibit hyperhomocysteinemia and decreased methylation capacity, with neuropathology and aortic lipid deposition. *Human molecular genetics* **2001**, *10*, 433-443.
59. Greig, K.T.; Antonchuk, J.; Metcalf, D.; Morgan, P.O.; Krebs, D.L.; Zhang, J.G.; Hacking, D.F.; Bode, L.; Robb, L.; Kranz, C., *et al.* Agm1/pgm3-mediated sugar nucleotide synthesis is essential for hematopoiesis and development. *Molecular and cellular biology* **2007**, *27*, 5849-5859.
60. Wang, L.; Magdaleno, S.; Tabas, I.; Jackowski, S. Early embryonic lethality in mice with targeted deletion of the ctp:Phosphocholine cytidylyltransferase alpha gene (pcyt1a). *Molecular and cellular biology* **2005**, *25*, 3357-3363.
61. Wu, G.; Aoyama, C.; Young, S.G.; Vance, D.E. Early embryonic lethality caused by disruption of the gene for choline kinase alpha, the first enzyme in phosphatidylcholine biosynthesis. *The Journal of biological chemistry* **2008**, *283*, 1456-1462.
