# Supplementary material for: Using Gene Essentiality and Synthetic Lethality Information to Correct Yeast and CHO Cell Genome-Scale Models
Source: Metabolites. 2015 Sep 29;5(4):536–70. doi: 10.3390/metabo5040536 (PMC4693185; doi:10.3390/metabo5040536)
Supplement: Supplementary File 1 [file metabolites-05-00536-s001.zip › S2.pdf]

Supplemental File 2:

Using gene essentiality and synthetic lethality information to correct Yeast and CHO cell genome-scale models

Authors: Ratul Chowdhury, Anupam Chowdhury, and Costas D. Maranas

Department of Chemical Engineering, The Pennsylvania State University, University Park, PA

Mathematical description of the Precursor Identifier for identifying blocked biomass precursors in as a result of *in silico* gene deletions

Removal of an essential reaction (or a synthetic lethal combination of reactions) in a metabolic network impairs the network's ability to channel substrate flux towards one or more metabolite precursors present in the representative biomass reaction of the genome-scale model. It is often necessary to first identify the blocked biomass precursor in order to understand the mechanism in which a reaction removal impairs cellular growth. As an alternative to time-consuming manual curation, the Precursor Identifier algorithm describes a computational method of systematically identifying all the biomass precursors that are blocked in the model as a result of reaction removals. In this procedure, we iterate through each precursor metabolite at a time, and modify the biomass equation such that the queried metabolite is the sole constituent of biomass. The model is incapable of synthetizing a particular precursor if the maximum flux of the corresponding modified biomass reaction is zero on solving a Flux Balance Analysis problem. Alternately, when none of the biomass precursors are blocked, the precursor for which flux through modified biomass equation is least is the limiting precursor determining the maximum achievable biomass flux in the network. In addition to its application in essentiality and synthetic lethality analyses, this procedure can be used during reconstruction of genome-scale models to find gaps in the metabolic network.

The description of the algorithm requires the definition of the following. To this end, we first define the following sets, variables and parameters:

Sets

$I = \{i \mid i = 1,2,...,N\} =$ set of metabolites
$J = \{j \mid j = 1,2,...,M\} =$ set of reactions
$PREC_i =$ set of metabolites present in the biomass equation

where, N and M signify total number of unique metabolites and reactions in the network respectively.

Variables

$v_j =$ flux through reaction j

Parameters

$S_{ij} =$ stoichiometric coefficient of metabolite i in reaction j
$LB_j =$ lower bound of flux through reaction j
$UB_j =$ upper bound of flux through reaction j
$M_{i^*i} =$ binary matrix containing information of the biomass precursor i^* and its associated metabolite present in the biomass equation, where $i^* \in PREC_i$

An inspection of the biomass reaction reveals that it constitutes of two types of precursors: (i) metabolite such as 1,3 betaD-glucan (13BDG) and 1,6 betaD-glucan that are unassociated with any product metabolite, and (ii) metabolites associated with a product metabolite synthesized in the biomass reaction. For example, the charged tRNA for arginine (Arg-tRNA(Arg)) is a precursor that releases the uncharged tRNA(Arg) in the biomass equation. This uncharged tRNA(Arg) is recycled back to be used in the Arg-AtRNA ligase reaction. For these precursors it is necessary to include both the reactant and the associated product in the modified biomass equation when performing the Precursor Analysis to obtain meaningful results. As a result, we define a matrix M_{i*i} that contains the information of all the metabolites that should be present in the modified biomass equation when performing the analysis for precursor $i*$ (for all $i* \in PREC_i$). In case of 13BDG, M is equal to a value of one only for itself and zero for all other metabolites. On the contrary, for Arg-tRNA(Arg) M is equal to a value of one both for itself and tRNA(Arg), while zero for all other metabolites.

The Precursor Identifier algorithm can be formulated as follows:

$$
\left[\begin{array}{lll}
\underset{v_j}{Maximize} \quad v_{biomass} & & \\
s.t & & \\
\sum_j S_{ij} v_j = 0, & \forall i \in I & (1) \\
S_{i,biomass} = S_{i,biomass} * M_{i^* i}, & \forall i \in I & (2) \\
LB_j \le v_j \le UB_j, & \forall j \in J & (3)
\end{array}\right] \quad \forall i^* \in PREC_i
$$

Here, we maximize the flux through the modified biomass reaction while iterating through each biomass precursor $i*$. The stoichiometry matrix remains unchanged for all reactions in the model except for the biomass reaction (Constraint 1). Constraint 2 modifies the biomass reaction such that it consists of only current precursor $i*$ at the time of iteration with any product metabolite associated with it. Constraint 1 enforces the steady-state stoichiometric balance in the network while constraint 3 sets the bounds of the fluxes for each reaction. Note that the information of any reaction removal can be implemented in the model by setting its LB and UB to zero. Metabolites in $PREC_i$ for which the maximum biomass flux is zero are blocked as a result of the removal of an essential reaction or their synthetic lethal combinations.
