# Supplementary material for: Using Gene Essentiality and Synthetic Lethality Information to Correct Yeast and CHO Cell Genome-Scale Models
Source: Metabolites. 2015 Sep 29;5(4):536–70. doi: 10.3390/metabo5040536 (PMC4693185; doi:10.3390/metabo5040536)
Supplement: Supplementary File 1 [file metabolites-05-00536-s001.zip › S4.pdf]

Supplemental File 4: Supplementary *in silico* and *in vivo* information on Yeast 7.11 and CHO 1.2

Table 1. Model modifications from Yeast 7.00 to Yeast 7.11 as reported in sourceforge.net

**Model modifications with reaction IDs of Yeast 7.11**	References (if any)
• Aspartate transport r_1117 can only flow in cytoplasm to mitochondria direction, to avoid free generation of proton gradient	Reported by Hnin Aung
• Malic enzyme r_0718 is NADP-dependent, not NAD^+	[1], [2], [3]
• NADH: ubiquinone oxidoreductase r_0773 is not proton translocating	[4], [5]
• ATP synthase r_0226 moves 4 cytoplasmic protons	NA
• Allow proton leak r_2129 from cytoplasm to mitochondrion	NA
• Mitochondrial ADP/ATP transporter r_1110 does not cotransport protons	[6], [7]
• Zymesterol intermediate 1c reactions r_0234, r_0240	[8]
• GDH2 irreversible	NA
• Remove citrate isomerase r_0303	NA
• Dihydoorotic acid dehydrogenase r_0453 uses only fumarate as electron donor: Remove r_0338, r_0339, r_2127	NA
• Pyruvate can be transported r_1254 out of the cell	[9]
• Folic acid is changed to Folate	NA
• Heme O monooxygenase r_0530 associated with genes (YPL252C AND YDR376W) or (YPL252C AND YDR376W AND YER141W)	NA
• Add chitin and heme a to biomass definition r_4041 with minimal stoichiometry (10^-6)	NA
• Allow potassium exchange r_2020 from medium	NA
• Extracellular reaction raffinose -> fructose + melibiose is catalysed by gene SUC2	[10]
• PHM8 is a 5'-nucleotidase for CMP r_0076, GMP r_1619 and UMP r_0078	[11]
• PNP1 phosphorylase activity specific to inosine and guanosine	[11]
• URH1 hydrolase activity specific to uridine	[11]
• PGM3 is a phosphoribomutase r_0907, not a phosphoglucomutase r_0888	[11]
• Metabolite formula, charge and number of carbon atoms added to SBML	Contributors: Hnin Aung, Kevin Correia, Ben Heavner, Kieran Smallbone, Balázs Szappanos

Table 2. Single gene deletion data sets; one showing *in silico* essential genes, which have been verified *in vivo* and the other showing the ones which are not *in vivo* essentials

**Experimentally verified list**	***in silico* essential but non- essential *in vivo***	**Experimentally verified list**	***in silico* essential but non- essential *in vivo***	**Experimentally verified list**	***in silico* essential but non- essential *in vivo***
YAR015W	YBL076C	YEL058W	YHR208W	YGL245W	
YBL033C	YBR115C	YER003C	YJL167W	YGR060W	
YCL018W	YBR153W	YER014W	YJR148W	YGR264C	
YDL055C	YBR166C	YER052C	YLL018C	YHR007C	
YDL103C	YBR248C	YER055C	YLR060W	YHR019C	
YDL141W	YBR256C	YER090W	YLR359W	YHR025W	
YDL205C	YBR265W	YFR025C	YLR420W	YHR072W	
YDR007W	YCL030C	YGL001C	YMR062C	YIL116W	
YDR037W	YDR044W	YGL009C	YMR300C	YIR034C	
YDR047W	YDR226W	YGL148W	YMR319C	YJL088W	
YDR234W	YER023W	YGL154C	YNL277W	YJL130C	
YDR487C	YFL017C	YGL234W	YOL143C	YJR139C	
YKL104C	YFL022C	YOR095C	YOR130C	YOR335C	
YKL211C	YFL045C	YOR128C	YGL055W	YPL160W	
YKL216W	YGR185C	YOR168W	YPL252C	YPL172C	

YLR100W	YOR176W	YPR033C
YLR304C	YOR236W	YPR035W
YLR355C	YOR278W	YPR060C
YMR220W	YPR183W	
YNL220W	YPL231W	
YNL280C	YLR303W	
YNR016C	YGR204W	
YMR202W	YBR126C	
YMR217W	YDR074W	
YNR050C	YJR109C	
YOL140W	YOR303W	
YOR074C	YPR167C	

Table 3. Double gene deletion data sets; one showing *in silico* SL gene pairs, which have been verified *in vivo* and the other showing the ones which have not been tested *in vivo*

**Experimentally verified list**	***in silico* predictions not tested *in vivo***
YBR117C, YPR074C	YAL054C, YLR153C
YBR249C, YDR035W	YBR294W, YLR092W
YGL125W, YPL023C	YDL131W, YDL182W
YGR143W, YPR159W	YML106W, YMR271C
YGR209C, YLR043C	YOR222W, YPL134C
YGL202W, YHR137W	YBR121C, YPR081C
YJL121C, YNL241C	YDR035W, YKL029C
YBL039C, YJR103W	YCL050C, YJR010W
YKL181W, YOL061W	YMR170C, YPL061W
YDR502C, YLR180W	YDR300C, YLR438W
YGR032W, YLR342W	YBR291C, YKL029C
YCR034W, YLR372W	YNL104C, YOR108W
YLR450W, YML075C	
YLR028C, YMR120C	
YGR124W, YPR145W	

Table 4. Higher order gene deletion data sets; one showing *in silico* SL gene sets, which have been verified *in vivo* and the other showing the ones which have not been tested *in vivo*

	**Experimentally verified list**	***in silico* predictions not tested *in vivo***
**SL Triplets**	YER099C, YHL011C, YBL068W	YCL040W, YFR053C, YGL253W
	YDL095W, YAL023C, YJR143C	YHR216W, YLR432W, YML056C
		YGR254W, YHR174W, YKR097W
		YAL062W, YDL171C, YOR375C
		YDR497C, YJL153C, YOL103W
**SL Quadruplets**	No information	YDR287W, YDR497C, YHR046C, YOL103W
	No information	YEL046C, YGR087C, YLR044C, YLR134W

Table 5. List of essential genes for the CHO 1.2 model along with auxotrophy information

Gene	Auxotrophy	Gene	Auxotrophy
acaCb	sphingomyelin	*lysXt*	L-lysine
acsL1	sphingomyelin	*metXt*	L-methionine
acsL2	sphingomyelin	*mthFr*	5-Methyltetrahydrofolate
acsL3	sphingomyelin	*mvd*	cholesterol
acsL4	sphingomyelin	*mvk*	cholesterol
akrLa4	sphingomyelin	*nadS*	NAD^+
aldOa1	phosphatidylglycerol<br>phosphatidylserine<br>phosphatidylethanolamine<br>phosphatidylcholine<br>cardiolipin<br>1Phosphatidyl-D-myoinositol	*nmnAt*	NAD^+, NADH, $NADP^+$
arg1	spermidine<br>putrescine	*odc1*	Spermidine<br>putrescine
argXt	L-arginine<br>spermidine<br>putrescine	*otrA41*	spermidine
cds1	L-arginine<br>spermidine<br>putrescine	*pinS*	1Phosphatidyl-D-myoinositol
crlS1	phosphatidylglycerol<br>1Phosphatidyl-D-myoinositol<br>cardiolipin	*pisD*	phosphotidylethanolamine
fasN	cholesterol	*pmvK*	cholesterol
ffa10	cholesterol	*qprT*	NAD^+, NADH, $NADP^+$, NADPH
ffa11	cholesterol	*sgmS1*	glycogen
ffa12	cholesterol	*sptLc1*	sphingomyelin
ffa7	cholesterol	*sptLc2*	sphingomyelin
ffa9	cholesterol	*sptLc3*	sphingomyelin
fvt1	sphingomyelin	*sqlE*	cholesterol
ggpS1	cholesterol	*srm*	spermidine
gys1	glycogen	*thrXt*	L-threonine
gys2	glycogen	*trpXt*	L-tryptophan
haaO	NAD+, NADH, NADP+, NADPH	*ugp2*	glycogen
hisXt	histidine	*valXt*	valine
hmgCr	cholesterol	*fdfT1*	sphingomyelin
idi1	cholesterol	*ebp*	cardiolipin
ileXt	L-isoleucine	*afmId*	sphingomyelin
khk	phosphatidylglycerol<br>phosphatidylserine<br>phosphatidylethanolamine<br>phosphatidylcholine<br>cardiolipin<br>1Phosphatidyl-D-myoinositol		

Table 6. List of SL gene pairs for the CHO 1.2 model along with auxotrophy information

Gene Pair	Auxotrophy	Gene Pair	Auxotrophy
asnS, asnXt	L-asparagine	*pemT, slc5a7*	sphingomyeline<br>phoshphatidylcholine
pbeF1, prpS2	NAD+, NADP+, NADPH	*dhcR24, sc5D*	cholesterol
pc3, ahcYl1	phosphatidylcholine	*isyNa1, ugtLa2*	1Phosphatidyl-D-myoinositol
pc4, slc5a7	sphingomyeline, phoshphatidylcholine	*chkB, ptdSs1*	sphingomyelin<br>phosphatidylserine<br>phosphatidylethanolamine<br>phosphatidylcholine
ahcYl1, slc5a7	sphingomyeline, phoshphatidylcholine	*ptdSs1, pcyT2*	phosphatidylserine<br>phosphatidylethanolamine
pc3, pc4	phoshphatidylcholine	*pc3, pc2*	phosphatidylserine<br>phosphatidylethanolamine<br>phosphatidylcholine
txnd2, tr3	dATP, dGTP, dCTP, dTTP	*impA2, mioX*	1Phosphatidyl-D-myoinositol
pc3, pcyT2	phoshphatidylcholine<br>phoshphatidylethanolamine<br>phoshphatidylserine	*isyNa1, gusB*	1Phosphatidyl-D-myoinositol
pheXt, tyrXt	L-phenylalanine, L-tyrosine	*polA1, tymS*	dTTP
pah, pheXt	L-phenylalanine	*impA2, ugtLa2*	1Phosphatidyl-D-myoinositol
slc14a2, slc14a1	spermidine, putrescine	*isyNa1, mioX*	1Phosphatidyl-D-myoinositol
dhcR24, choL4	cholesterol	*isyNa1, ugdH*	1Phosphatidyl-D-myoinositol
qdpR, pheXt	L-phenylalanine	*pc5, slc5a7*	sphingomyelin,<br>phosphatidylcholine
pc3, chkB	phosphatidylcholine<br>phosphatidylethanolamine	*impA2, ugdH*	1Phosphatidyl-D-myoinositol
pc2, ptdSs1	phosphatidylserine<br>phosphatidylethanolamine	*pc3, pc5*	phosphatidylcholine
fh1, sdhc	sphingomyeline	*choL4, sc5D*	cholesterol
chkA, chkB	sphingomyelin<br>phosphatidylserine<br>phosphatidylethanolamine<br>phosphatidylcholine	*pc3, pemT*	phosphatidylcholine
sdhD , sdhc	sphingomyelin	*cel, lpl*	phosphatidylglycerol<br>phosphatidylserine<br>phosphatidylethanolamine<br>phosphatidylcholine<br>cardiolipin<br>1Phosphatidyl-D-myoinositol
ptdSs1 , ptdSs2	phosphatidylserine<br>phosphatidylethanolamine	*indOl, tdo2*	NAD+, NADP+, NADPH
dtyMk , polA1	dTTP	*pcyT1a, pcyT1b*	sphingomyelin
gusB , impA2	1Phosphatidyl-D-myoinositol	*pgm1, pgm2*	glycogen
pc3 , ptdSs2	phosphatidylserine<br>phosphatidylethanolamine<br>phosphatidylcholine		

Table 7. List of higher order SL gene sets for the CHO 1.2 model along with auxotrophy information

	Higher order SL-gene sets	Auxotrophy
Synthetic Lethal Triplets	*dhoDh , fh1 , cytB*	*Sphingomyelin*
	ggh , pipOx , slc19a1	*5Methyltetrahydrofolate*
	cox1 , dhoDh , sdhD	*Sphingomyelin*
	cox2 , dhoDh , sdhD	*Sphingomyelin*
	cox3 , dhoDh , sdhD	*Sphingomyelin*
	cox5a , dhoDh , sdhD	*Sphingomyelin*
	cox5b , dhoDh , sdhD	*Sphingomyelin*
	cox6c , dhoDh , sdhD	*Sphingomyelin*
	cox7b , dhoDh , sdhD	*Sphingomyelin*
	cox8a , dhoDh , sdhD	*Sphingomyelin*
	cox7c , dhoDh , sdhD	*Sphingomyelin*
	cox8b , dhoDh , sdhD	*Sphingomyelin*
	cox6a1 , dhoDh , sdhD	*Sphingomyelin*
	cox6a2 , dhoDh , sdhD	*Sphingomyelin*
	cox6b1 , dhoDh , sdhD	*Sphingomyelin*
	cox7a1 , dhoDh , sdhD	*Sphingomyelin*
	cox6b2 , dhoDh , sdhD	*Sphingomyelin*
	cox7a2 , dhoDh , sdhD	*Sphingomyelin*
	cox7b2 , dhoDh , sdhD	*Sphingomyelin*
	cox7a2l , dhoDh , sdhD	*Sphingomyelin*
SL Quadruplets	*nanS , npl , st8Sia1 , st8Sia5*	*N-Acetylneuraminate*
	ak1 . ak2 , ak3L1 , cmpK1	*Phosphatidylglycerol*<br>*Phosphatidylserine*<br>*Phosphatidylethanolamine*<br>*Phosphatidylcholine*<br>*Cardiolipin*<br>*1Phosphatidyl-D-myoinositol*
	polA1 , rrm1, rrm2 , rrm2b	*dATP*

Table 8. List of gene members for the most important gene-hubs based on node centrality analysis

**Genes forming SL2 with *pc3***	**Genes forming SL2 with *slc5a7***	**Genes forming SL2 with *ptdSs1***	**Genes forming SL2 with *isyNa1***	**Genes forming SL2 with *chkB***	**Gene pair forming SL3 with *dhoDh* only**	**Genes forming SL3 with *sdhD - dhoDh* gene pair**
ahcYl1	*pc4*	*chkB*	*ugdH*	*pc3*	*cytB-fh1*	*cox1*
pc4	*ahcYl1*	*ptdSs2*	*gusB*	*chkA*		*cox2*
pc5	*pemT*	*pcyT2*	*mioX*	*ptdSs1*		*cox3*
pemT	*pc5*	*pc2*	*ugtLa2*			*cox5a*
pcyT2						*cox5b*
pc2						*cox6c*
chkB						*cox7b*
ptdSs2						*cox8a*
						cox8b
						cox7c
						cox6a1
						cox6a2
						cox6b1
						cox7a1
						cox6b2
						cox7a2
						cox7b2
						cox7a2l

1. Boles E, de Jong-Gubbels P, Pronk JT: **Identification and characterization of MAE1, the Saccharomyces cerevisiae structural gene encoding mitochondrial malic enzyme**. *Journal of bacteriology* 1998, **180**(11):2875-2882.
2. Moreira dos Santos M, Raghevendran V, Kotter P, Olsson L, Nielsen J: **Manipulation of malic enzyme in Saccharomyces cerevisiae for increasing NADPH production capacity aerobically in different cellular compartments**. *Metabolic engineering* 2004, **6**(4):352-363.
3. Suga H, Matsuda F, Hasunuma T, Ishii J, Kondo A: **Implementation of a transhydrogenase-like shunt to counter redox imbalance during xylose fermentation in Saccharomyces cerevisiae**. *Applied microbiology and biotechnology* 2013, **97**(4):1669-1678.
4. Velazquez I, Pardo JP: **Kinetic characterization of the rotenone-insensitive internal NADH: ubiquinone oxidoreductase of mitochondria from Saccharomyces cerevisiae**. *Archives of biochemistry and biophysics* 2001, **389**(1):7-14.
5. Dujon B: **Yeast evolutionary genomics**. *Nature reviews Genetics* 2010, **11**(7):512-524.
6. Klingenberg M: **The ADP-ATP translocation in mitochondria, a membrane potential controlled transport**. *The Journal of membrane biology* 1980, **56**(2):97-105.
7. Vozza A, Blanco E, Palmieri L, Palmieri F: **Identification of the mitochondrial GTP/GDP transporter in Saccharomyces cerevisiae**. *The Journal of biological chemistry* 2004, **279**(20):20850-20857.
8. Alvarez-Vasquez F, Riezman H, Hannun YA, Voit EO: **Mathematical modeling and validation of the ergosterol pathway in Saccharomyces cerevisiae**. *PloS one* 2011, **6**(12):e28344.
9. Zampar GG, Kummel A, Ewald J, Jol S, Niebel B, Picotti P, Aebersold R, Sauer U, Zamboni N, Heinemann M: **Temporal system-level organization of the switch from glycolytic to gluconeogenic operation in yeast**. *Molecular systems biology* 2013, **9**:651.
10. Gascon S, Neumann NP, Lampen JO: **Comparative study of the properties of the purified internal and external invertases from yeast**. *The Journal of biological chemistry* 1968, **243**(7):1573-1577.
11. Xu YF, Letisse F, Absalan F, Lu W, Kuznetsova E, Brown G, Caudy AA, Yakunin AF, Broach JR, Rabinowitz JD: **Nucleotide degradation and ribose salvage in yeast**. *Molecular systems biology* 2013, **9**:665**.**
