# Supplementary material for: Using Gene Essentiality and Synthetic Lethality Information to Correct Yeast and CHO Cell Genome-Scale Models
Source: Metabolites. 2015 Sep 29;5(4):536–70. doi: 10.3390/metabo5040536 (PMC4693185; doi:10.3390/metabo5040536)
Supplement: Supplementary File 1 [file metabolites-05-00536-s001.zip › S5.pdf]

Supplemental File 5:

The gap in the SL-2 landscape due to absence of *YDL040C* and *YMR307C*

The absence of the *YDL040C* and *YMR307C* genes in the Yeast 7.11 consensus reconstruction incapacitates the model to identify nine more *in vivo* synthetic lethal gene pairs as shown in Figures 1A and 1B (with the Y-id of genes from SGD and commercial gene names) respectively.

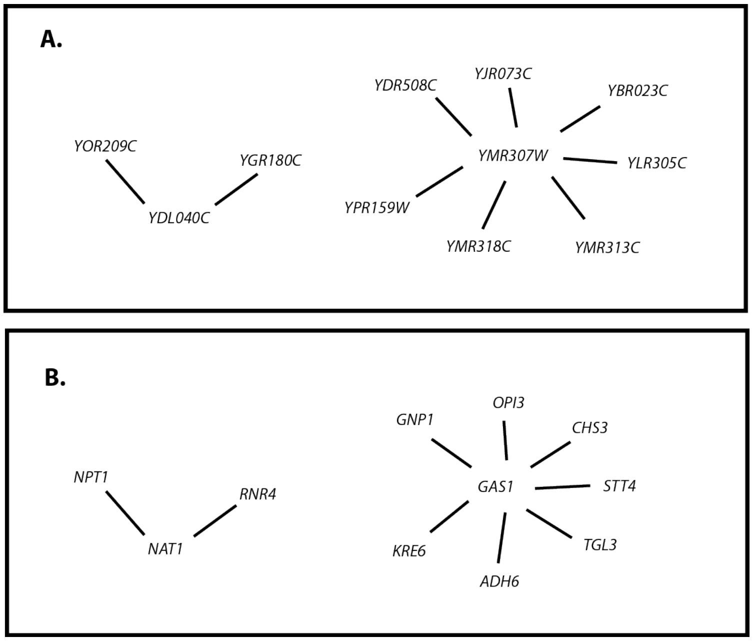

Figure 1. Lethal interactions missing in the model

YDL040C (NAT1)

Δnat1Δnpt1 strain

Pan X *et al* [1] reveals that S288C yeast strain under minimal media when subjected to double gene deletion, the resultant ***Δnat1Δnpt1*** strain is inviable. *NAT1* gene encodes the functional subunit of the N-terminal acetyltransferase (NAT) NatA protein responsible for cellular processes such as, cell cycle, heat-shock resistance, sporulation and telomeric silencing. It is also learnt [2] that *ARD1* and *NAT1* are structural subunits of the constitutive protein NAT1m, where *NAT1* encodes the catalytic subunit but *ARD1* encoded protein functionality is unclear. It has however been seen that both these subunits form homo-dimers in the absence of the other and when unable to form the functional hetero-dimer. N-terminal acetylation of most yeast proteins has shown 20-fold increase [3] in acetylation activity that indicates a suggestive GPR relation between them could be *NAT1 and ARD1*. *NAT1* and *ARD1* double deletion strains have reduced NAT activity and silent mating type locus HML.

Additionally, *NPT1* gene encodes nicotinate phosphoribosyltransferase that makes NAD+ biosynthesis conducive. Besides, this nuclear protein is also active in rDNA and telomere silencing. There exists no *in vivo* information as to why is the double mutant lethal. Figure 2 show that ***Δnpt1*** mutants can restore Sir2- dependent recombination defects [4, 5] when allowed to uptake NAD+ precursor riboside. It has also been reported that NAD+ salvage enzyme *NPT1* associates with heterochromatic regions besides contributing to growth regulation via NAD+ availability and synthesis in yeast cells.

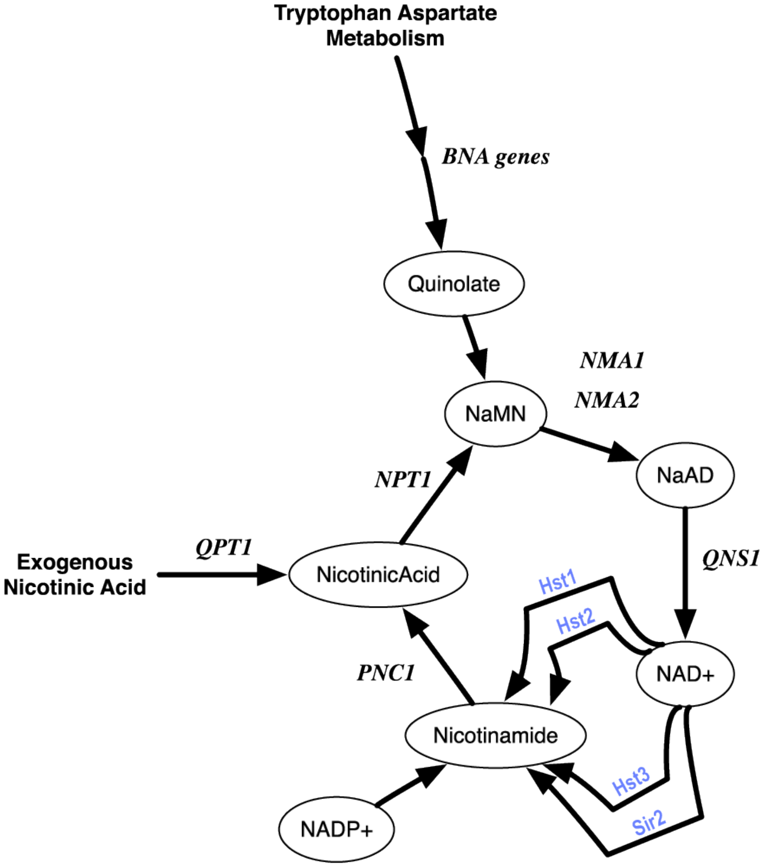

Figure 2 NPT1 gene importance for NAD+ production

Due to unavailability of the full telomere-silencing pathway inside the yeast reconstruction, the current model cannot capture this synthetic lethal pair. Even though it might appear that telomere silencing is a purely non-metabolic phenomenon, works such as Rossmann MP *et al* [6] reveal that it is associated with nucleotide metabolism. This integration is beyond the scope of this paper and calls for an interesting future work towards updating the current reconstruction. However, we predict that a ***ΔNAT1ΔNPT1*** mutant strain would be an NAD+ auxotroph and hence inviable. It is also interesting to note that *E. coli* and *C. acetobutylicum* genome scale models, peptides such as Ala-Ala, Ala-Thr, Ala-Ser are also involved in the murine biosynthesis pathway and participate in glucan biosynthesis for cell wall formation. *NAT1* gene is involved in acetylation of such peptides. However, Keppler-Ross *et al* [7] shows that yeast uses mannan instead of glucan for cell wall formation so a parallel cannot be drawn between yeast and other well-annotated prokaryotes along these lines.

ΔNAT1ΔRNR4 strain

*RNR4 (*ribonucleotide-diphosphate reductase) encodes a small subunit of the RNR $\alpha_2\beta_2$ protein tetramer complex that catalyzes the rate-limiting step in dNTP synthesis. Zhao *et al* [8] shows that null mutant of *RNR4* is inviable for W303 yeast strain but S288C is viable and sick [9]. Pan X *et al* [1] reveals the synthetic lethality of ***ΔNAT1ΔRNR4*** double mutant strain.

It can be summarized that a double mutant strain would be a dNTP auxotroph. The current reconstruction enlists dNDP and dNTP synthesis as catalyzed by the RNR complex (composed of subunits encoded by *RNR1, RNR2, RNR3, RNR4* genes). Figure 3 shows the composition and the functional behavior of RNR subunits as entailed by [10] and [11]. Domkin *et al* [11] shows the cross-talk and association between RNR1 and RNR3 subunits. Studies on active site of RNR4 subunits reveal its inability to bind iron and that it stabilizes the RNR2 subunit for cofactor assembly and activity [12]. It has been shown that ***ΔNAT1ΔRNR4*** is a lethal mutant however neither of *ΔNAT1ΔRNR1, ΔNAT1ΔRNR2, ΔNAT1ΔRNR3* are lethal [1]. This puts the existing GPR to question under the circumstances that the other subunits are unable to take over the functionality of RNR4. So the proposed GPR for the reactions for dNTP biosynthesis should be

(*RNR4 or (RNR1 and RNR2 and RNR3 and RNR4*)). However, due to absence of additional proofs we do not include this change as a part of our model modifications.

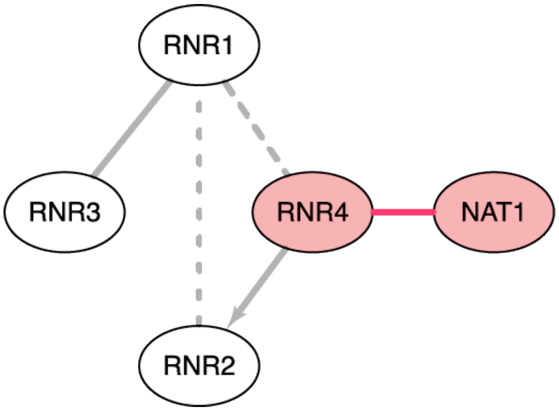

Figure 3 The grey lines represent the functional interaction of the RNR subunits in the RNR tetramer complex. The red interacting genes show synthetic lethal gene interaction between *RNR4* and *NAT1* gene. It is understood that there is profound cross talk between RNR3 and RNR1 subunits. Again, it has been shown by the arrowhead that RNR4 is key to activation of RNR2's catalytic site. It is interesting to see that RNR1-RNR2 and RNR1-RNR4 have dotted lines representing that interaction of RNR2 and RNR4 with RNR1 is sterically space limited, which means, either RNR2 and RNR4 gets to bind with RNR1. This is supported by the fact that RNR2 is a homolog from whole gene duplication of RNR4 and bears over 80% sequence similarity. However, RNR4 needs to activate RNR2 for it to function.

Conclusion

These informations can be used in subsequent yeast models to capture the missing lethal genetic interactions.

YMR307W (GAS1)

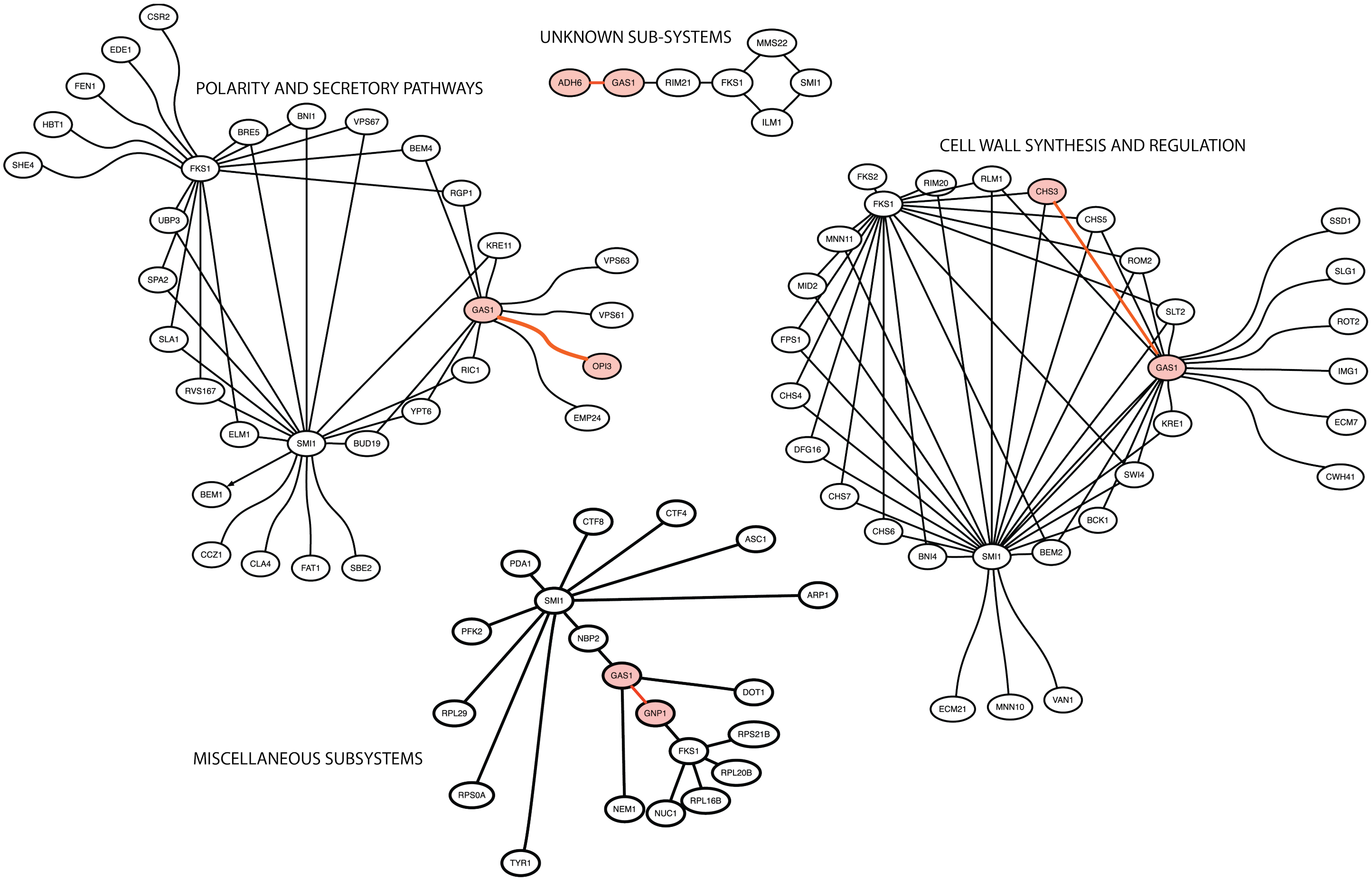

Figure 4 Shows the lethality landscape of lethal pairs *in vivo*. The sub-landscapes reveal the node degree and for the gene in question *GAS1*. This figure aims to show four of the lethal pairs of *GAS1, viz. OPI3, ADH6, CHS3* and GNP1.

GAS1 gene

GNP1 is key to *in vivo* lethal pair genetic clusters (see Fig 4). Marked in red are the genetic interactions we have noted in Figure 1B. However, due to the ambiguous nature of the subsystems of which these interactions are a part of, we conclude that it is not possible to restore the lethal interactions in the current reconstruction. Nevertheless, it still opens an avenue to curate models using lethality information, if subsequent genome scale models can incorporate pathways pertaining to cell polarity, cell-wall synthesis and its regulation.

1. Pan X, Ye P, Yuan DS, Wang X, Bader JS, Boeke JD: **A DNA integrity network in the yeast Saccharomyces cerevisiae**. *Cell* 2006, **124**(5):1069-1081.
2. Park EC, Szostak JW: **ARD1 and NAT1 proteins form a complex that has N-terminal acetyltransferase activity**. *The EMBO journal* 1992, **11**(6):2087-2093.
3. Mullen JR, Kayne PS, Moerschell RP, Tsunasawa S, Gribskov M, Colavito-Shepanski M, Grunstein M, Sherman F, Sternglanz R: **Identification and characterization of genes and mutants for an N-terminal acetyltransferase from yeast**. *The EMBO journal* 1989, **8**(7):2067-2075.
4. Belenky P, Bogan KL, Brenner C: **NAD+ metabolism in health and disease**. *Trends in biochemical sciences* 2007, **32**(1):12-19.
5. Sandmeier JJ, Celic I, Boeke JD, Smith JS: **Telomeric and rDNA silencing in Saccharomyces cerevisiae are dependent on a nuclear NAD(+) salvage pathway**. *Genetics* 2002, **160**(3):877-889.
6. Rossmann MP, Luo W, Tsaponina O, Chabes A, Stillman B: **A common telomeric gene silencing assay is affected by nucleotide metabolism**. *Molecular cell* 2011, **42**(1):127-136.
7. Keppler-Ross S, Douglas L, Konopka JB, Dean N: **Recognition of yeast by murine macrophages requires mannan but not glucan**. *Eukaryotic cell* 2010, **9**(11):1776-1787.
8. Zhao X, Muller EG, Rothstein R: **A suppressor of two essential checkpoint genes identifies a novel protein that negatively affects dNTP pools**. *Molecular cell* 1998, **2**(3):329-340.

9. Wang A, Forman-Kay J, Luo Y, Luo M, Chow YH, Plumb J, Friesen JD, Tsui LC, Heng HH, Woolford JL, Jr. *et al*: **Identification and characterization of human genes encoding Hprp3p and Hprp4p, interacting components of the spliceosome**. *Human molecular genetics* 1997, **6**(12):2117-2126.
10. Xu H, Faber C, Uchiki T, Racca J, Dealwis C: **Structures of eukaryotic ribonucleotide reductase I define gemcitabine diphosphate binding and subunit assembly**. *Proceedings of the National Academy of Sciences of the United States of America* 2006, **103**(11):4028-4033.
11. Domkin V, Thelander L, Chabes A: **Yeast DNA damage-inducible Rnr3 has a very low catalytic activity strongly stimulated after the formation of a cross-talking Rnr1/Rnr3 complex**. *The Journal of biological chemistry* 2002, **277**(21):18574-18578.
12. Sommerhalter M, Voegtli WC, Perlstein DL, Ge J, Stubbe J, Rosenzweig AC: **Structures of the yeast ribonucleotide reductase Rnr2 and Rnr4 homodimers**. *Biochemistry* 2004, **43**(24):7736-7742.
