# Supplementary material for: Using Gene Essentiality and Synthetic Lethality Information to Correct Yeast and CHO Cell Genome-Scale Models
Source: Metabolites. 2015 Sep 29;5(4):536–70. doi: 10.3390/metabo5040536 (PMC4693185; doi:10.3390/metabo5040536)
Supplement: Supplementary File 1 [file metabolites-05-00536-s001.zip › S6.pdf]

Supplemental File 6:
Comparison of Mouse and CHO lethal gene sets

The SL pairs in mouse genome scale model (139 gene pairs)

Gene 1- Gene 2 refer to a synthetic lethal pair

GENE 1	GENE2	GENE1	GENE2	GENE1	GENE2	GENE1	GENE2
AAPR	*GPAT1*	*FFAM06*	*FFAM16*	*MIPL*	*UGDH*	*PGLS*	*TRALD*
ADSLa	*MTHFD1*	*FFAM15*	*FFB05*	*MIPL*	*UGT2B5*	*PHEXT*	*TYRXT*
ADSLa	*MTHFD2a*	*FFAM16*	*FFB05*	*MTHFD1*	*PAICS*	*POLA11*	*RRM1*
ADSLa	*MTHFD2b*	*FFAM19*	*FFB03*	*MTHFD1*	*PAICSa*	*POLA13*	*RRM2b*
ADSLa	*SLC26A6*	*FFB01*	*FFB47*	*MTHFD1*	*PFAS*	*POLA14*	*TMK*
AFMIDa	*AFMIDc*	*FFB03*	*FFE03*	*MTHFD1*	*PRPS2*	*POLA14*	*TYMS*
AFMIDc	*KYNUa*	*FFB04*	*FFB51*	*MTHFD1*	*PURH*	*PRPS2*	*SLC26A6*
AFMIDc	*KYNUb*	*Fha*	*MTHFD1*	*MTHFD1*	*PURHa*	*PSS1*	*PSS2*
AGPAT	*CELb*	*Fha*	*MTHFD2a*	*MTHFD2a*	*PAICS*	*PURH*	*SLC26A6*
AGPAT	*HIBCHb*	*Fha*	*MTHFD2b*	*MTHFD2a*	*PAICSa*	*PURHa*	*SLC26A6*
AGPAT	*HPG22*	*Fha*	*SLC26A6*	*MTHFD2a*	*PFAS*	*SDHC*	*SUDCb*
AGPAT	*HPG33*	*FHb*	*SUDCb*	*MTHFD2a*	*PRPS2*	*SLC14A1*	*SLC14A2*
ASNS	*ASNXT*	*FTHFL*	*MTHFD1*	*MTHFD2a*	*PURH*	*TR3b*	*TXNRD2*
CBSa	*CYSXT*	*FTHFL*	*MTHFD2a*	*MTHFD2a*	*PURHa*		
CBSa	*MTR*	*FTHFL*	*MTHFD2b*	*MTHFD2b*	*PAICS*		
CHK	*CHKL*	*FTHFL*	*SLC26A6*	*MTHFD2b*	*PAICSa*		
CHOK	*PC3*	*GARc*	*MTHFD1*	*MTHFD2b*	*PFAS*		
CHOK	*PSS1*	*GARc*	*MTHFD2a*	*MTHFD2b*	*PRPS2*		
CHOL4	*DHCR24b*	*GARc*	*MTHFD2b*	*MTHFD2b*	*PURH*		
CHOL4	*EBPa*	*GARc*	*SLC26A6*	*MTHFD2b*	*PURHa*		
CHOL4	*HPG09*	*GARTa*	*MTHFD1*	*MTR*	*MUTa*		
CHOL4	*HPG10*	*GARTa*	*MTHFD2a*	*MTR*	*PCCB*		
CHOL4	*SC5D*	*GARTa*	*MTHFD2b*	*NADK*	*NT5M6*		
CHOL5	*DHCR24b*	*GARTa*	*SLC26A6*	*OGDH2*	*SUDCb*		
CHOL5	*EBPa*	*GARTb*	*MTHFD1*	*OGDH3*	*SUDCb*		
CHOL5	*HPG09*	*GARTb*	*MTHFD2a*	*PAHb*	*PHEXT*		
CHOL5	*HPG10*	*GARTb*	*MTHFD2b*	*PAICS*	*SLC26A6*		
CHOL5	*SC5D*	*GARTb*	*SLC26A6*	*PAICSa*	*SLC26A6*		
CTHc	*CYSXT*	*GDC1*	*GYK*	*PBEF1*	*RPPK*		
CTHc	*MTR*	*GPAT1*	*GPAT2*	*PC1*	*PC3*		
CYSOUT	*MTR*	*GPD2*	*RPE*	*PC1*	*PSS1*		
CYSXT	*HPG25*	*GPD2*	*TKT*	*PC2*	*PC3*		
CYSXT	*MUTa*	*GPD2*	*TKTa*	*PC2*	*PSS1*		
CYSXT	*PCCB*	*GPD2*	*TRALD*	*PC3*	*PSS2*		
DHCR24a	*SC5D*	*GUSBa*	*IMPA2*	*PFAS*	*SLC26A6*		
DHPR1	*PHEXT*	*GUSBa*	*MIPL*	*PGD*	*RPE*		
DLST1	*SUDCb*	*HMGCLa*	*MGCH*	*PGD*	*TKT*		
FDFT1a	*FDFT1b*	*HPG25*	*MTR*	*PGD*	*TKTa*		
FDFT1a	*FDFT1c*	*IMPA2*	*MIOX*	*PGD*	*TRALD*		
FFAM04	*FFAM19*	*IMPA2*	*UGDH*	*PGLS*	*RPE*		
FFAM04	*FFE03*	*IMPA2*	*UGT2B5*	*PGLS*	*TKT*		
FFAM06	*FFAM15*	*MIOX*	*MIPL*	*PGLS*	*TKTa*		

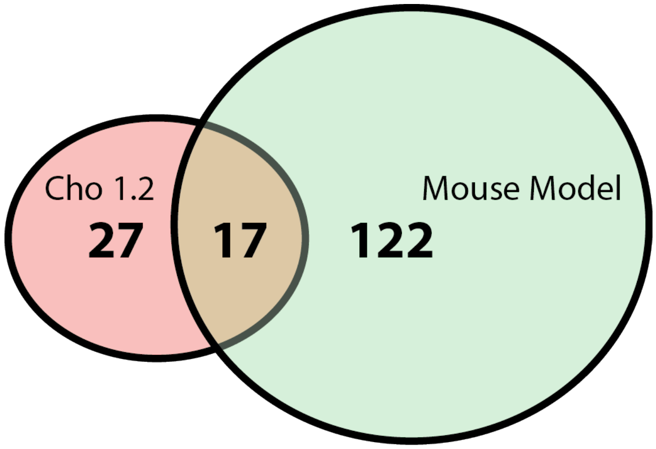

Figure: Overlap of mouse and CHO 1.2 synthetic lethal pairs

Table: Showing the common SL pairs between CHO 1.2 and mouse GSM

The SL pairs COMMON in mouse genome scale model and CHO 1.2

Gene 1- Gene 2 refers to a synthetic lethal pair

GENE 1	GENE2
asnS	*asnXt*
pheXt	*tyrXt*
pah	*pheXt*
ptdSs1	*ptdSs2*
gusB	*impA2*
pc3	*ptdSs2*
dhcR24	*sc5D*
pc3	*pc2*
impA2	*mioX*
polA1	*tymS*
impA2	*ugdH*
choL4	*sc5D*
txnd2	*tr3*
slc14a2	*slc14a1*
dhcR24	*choL4*
pc2	*ptdSs1*
chkA	*chkB*
